# Remodeling Serine Synthesis and Metabolism via Nanoparticles (NPs)‐Mediated CFL1 Silencing to Enhance the Sensitivity of Hepatocellular Carcinoma to Sorafenib

**DOI:** 10.1002/advs.202207118

**Published:** 2023-05-18

**Authors:** Senlin Li, Lei Xu, Guo Wu, Ziqi Huang, Linzhuo Huang, Fengqian Zhang, Chunfang Wei, Qian Shen, Rong Li, Lei Zhang, Xiaoding Xu

**Affiliations:** ^1^ Guangdong Provincial Key Laboratory of Malignant Tumor Epigenetics and Gene Regulation Guangdong‐Hong Kong Joint Laboratory for RNA Medicine Medical Research Center Sun Yat‐Sen Memorial Hospital Sun Yat‐Sen University Guangzhou 510120 P. R. China; ^2^ Nanhai Translational Innovation Center of Precision Immunology Sun Yat‐Sen Memorial Hospital Foshan 528200 P. R. China; ^3^ Department of Hepatobiliary Surgery Sun Yat‐Sen Memorial Hospital Sun Yat‐Sen University Guangzhou 510120 P. R. China; ^4^ The Second Affiliated Hospital Hengyang Medical School University of South China Hengyang 421001 P. R. China

**Keywords:** cofilin 1, hepatocellular carcinoma, nanoparticles, serine synthesis and metabolism, sorafenib sensitivity

## Abstract

Tyrosine kinase inhibitors represented by sorafenib are the first‐line treatment for hepatocellular carcinoma (HCC), but the low response rate of HCC patient has become a clinical pain‐point. Emerging evidences have revealed that metabolic reprogramming plays an important role in regulating the sensitivity of tumor cells to various chemotherapeutics including sorafenib. However, the underlying mechanisms are very complex and are not fully elucidated. By comparing the transcriptome sequencing data of sorafenib‐sensitive and ‐insensitive HCC patients, it is revealed that cofilin 1 (CFL1) is highly expressed in the tumor tissues of sorafenib‐insensitive HCC patients and closely correlated with their poor prognosis. Mechanically, CFL1 can promote phosphoglycerate dehydrogenase transcription and enhance serine synthesis and metabolism to accelerate the production of antioxidants for scavenging the excessive reactive oxygen species induced by sorafenib, thereby impairing the sorafenib sensitivity of HCC. To translate this finding and consider the severe side effects of sorafenib, a reduction‐responsive nanoplatform for systemic co‐delivery of CFL1 siRNA (siCFL1) and sorafenib is further developed, and its high efficacy in inhibiting HCC tumor growth without apparent toxicity is demonstrated. These results indicate that nanoparticles‐mediated co‐delivery of siCFL1 and sorafenib can be a new strategy for the treatment of advanced HCC.

## Graphical abstract

Herein, the important function of **cofilin 1 (CFL1)** in regulating sorafenib sensitivity of hepatocellular carcinoma (HCC) via enhancing serine synthesis and metabolism to accelerate antioxidant production for scavenging the excessive reactive oxygen species triggered by sorafenib is identified and demonstrated. Based on this regulatory mechanism, systemic co‐delivery of siCFL1 and sorafenib with reduction‐responsive nanoparticles can effectively silence CFL1 expression and significantly inhibit HCC patient‐derived xenografts tumor growth.

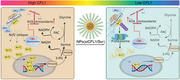

## 1 Introduction

Hepatocellular carcinoma (HCC) is the most common type of primary liver cancer and the third leading cause of cancer‐related deaths worldwide.^[^ ^1^, ^2^ ^]^ Sorafenib, a multiple tyrosine kinase inhibitor (TKI) that can target Raf kinases, vascular endothelial growth factor receptor, and platelet‐derived growth factor receptor and block their downstream signaling pathways, is currently the first‐line treatment for advanced and unresectable HCC.^[^ ^3^, ^4^ ^]^ However, clinical observations have shown that appropriately 70% of HCC patients are insensitive to sorafenib treatment.^[^ ^5^, ^6^ ^]^ Moreover, almost all the sorafenib‐sensitive patients will eventually develop drug resistance within 6 months.^[^ ^6^, ^7^ ^]^ Although the new generation of TKIs (e.g., regorafenib and lenvatinib) has been approved for clinical application, they are still not beneficial for the sorafenib‐insensitive HCC patients.^[^ ^8^ ^]^ Currently, Nivolumab, an immune checkpoint inhibitor, has been clinically used for advanced HCC treatment, but the low response rate and immune side effects remain a big challenge.^[^ ^9^ ^]^ Overall, the treatment options for advanced HCC patients are still very limited and exploring the key regulators contributing to sorafenib sensitivity could thus provide novel therapeutic targets and more insights for combination HCC therapy.

The underlying mechanisms regulating sorafenib sensitivity are very complex, in which autophagy, epigenetic modifications, epithelial‐mesenchymal transition, and the activation of compensatory tumor‐promoting pathways (e.g., Wnt/*β*‐catenin, PI3K/Akt, and MAPK/MEK) are widely involved.^[^ ^6^, ^10^ ^]^ In recent years, numerous evidences have revealed that metabolic reprogramming plays an important role in regulating the sensitivity of tumor cells to widely used first‐line chemotherapeutics.^[^ ^6^, ^11^ ^]^ One well‐recognized mechanism is that chemotherapeutics can induce the production of reactive oxygen species (ROS) via attacking the electron transport chain (ETC) complexes to amplify intracellular oxidative stress.^[^ ^12^ ^]^ To evade this oxidative stress‐induced cell death, tumor cells could adaptively enhance their metabolism especially amino acid synthesis and metabolism to accelerate the production of antioxidants for scavenging the excessive ROS and maintaining intracellular redox homeostasis.^[^ ^13^, ^14^, ^15^ ^]^ To explore the factors involved in the metabolic regulation of sorafenib sensitivity, a variety of drug‐resistant cells and animal models have been recently established and several key regulators have been identified as new therapeutic targets.^[^ ^14^, ^15^, ^16^ ^]^ However, the underlying mechanisms for metabolic regulation of sorafenib sensitivity are very complex and have not been fully elucidated. More importantly, emerging evidences have shown that the drug‐resistant cells and animal models may not reflect the real situation of clinical HCC patients due to the tumor complexity and heterogeneity.^[^ ^2^, ^17^ ^]^ In recent years, the use of high‐throughput sequencing technology to analyze clinical specimens has been demonstrated as an effective strategy to elucidate the underlying mechanisms for cancer progression including drug resistance and metastasis, showing the advantages of higher accuracy and better clinical significance.^[^ ^18^ ^]^

To get an in‐depth understanding of the mechanism regulating sorafenib sensitivity of HCC patients, we herein, for the first time, excavated the publicly available data of sorafenib‐sensitive (*n* = 21) and ‐insensitive tumor samples (*n* = 46) of HCC patients, and revealed that cofilin 1 (CFL1) is highly expressed in sorafenib‐insensitive HCC patients and closely correlated with the poor prognosis of HCC patients. Molecular mechanism study indicated that CFL1 could impair the interaction between Kelch‐like ECH‐associated protein 1 (Keap1) and erythroid 2‐related factor 2 (Nrf2) via promoting the depolymerization of filamentous actin (F‐actin) into globular actin (G‐actin) and improve the nuclear translocation of Nrf2 to enhance the transcription of phosphoglycerate dehydrogenase (PHGDH), which could accelerate the production of antioxidants via enhancing serine synthesis and metabolism to scavenge the excessive ROS triggered by sorafenib, thereby impairing the sensitivity of HCC cells to sorafenib. As a classic actin‐binding protein that can accelerate the turnover of actin filaments by depolymerizing F‐actin into G‐actin, CFL1 primarily involves the remodeling of cytoskeleton and cell motility.^[^ ^19^, ^20^ ^]^ Our work first uncovers the important role of CFL1 in regulating drug sensitivity. More importantly, considering the toxic and side effects of sorafenib^[^ ^7^, ^21^ ^]^ and the great potential of nucleic acid drugs (e.g., siRNA and mRNA) on cancer therapy,^[^ ^3^, ^22^ ^]^ we further constructed a reduction‐responsive nanoparticle (NP) platform to systematically co‐deliver CFL1 siRNA (siCFL1) and sorafenib, and demonstrated that this co‐delivery system could effectively enhance the sorafenib sensitivity of HCC cells via silencing CFL1 expression and significantly inhibit HCC tumor growth.

## 2 Results

### 2.1 CFL1 Is Highly Expressed in Sorafenib‐Insensitive HCC Patients and Indicates Poor Prognosis

**Figure 1:**
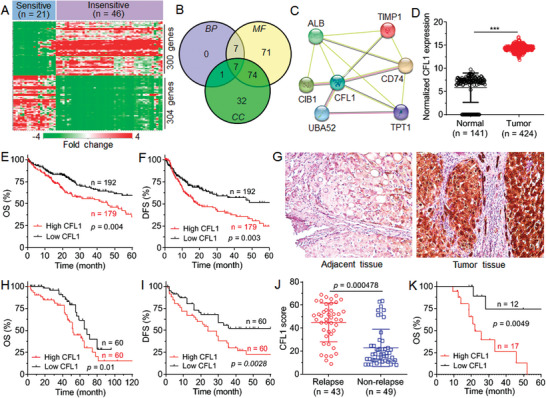
CFL1 is highly expressed in sorafenib‐insensitive HCC patients and indicates poor prognosis. A) mRNA expression profiles of tumor tissues of sorafenib‐sensitive (*n* = 21) and ‐insensitive HCC patients (*n* = 46) in GSE109211 dataset. B) The number of overlapped genes enriched in GO entries of biological processes (BP), cellular components (CC), and molecular function (MF). C) STRING analysis of protein–protein interaction network of seven candidate genes shown in (B). D) Gene expression profiling interactive analysis of CFL1 expression in normal liver tissues (*n* = 141) and tumor tissues (*n* = 424) of HCC patients in TCGA and GTEx database. *** *p* < 0.001. E,F) TCGA database showing the E) OS and F) DFS of HCC patients with different CFL1 expression levels. G) IHC staining analysis of CFL1 expression in adjacent and tumor tissues of one HCC patient. H) OS and I) DFS of HCC patients (*n* = 120) with different CFL1 expression levels. J) CFL1 score determined by IHC staining analysis of HCC patients with relapse (*n* = 43) or nonrelapse (*n* = 49) within postoperative 1 year. K) TCGA database showing the OS of HCC patients received standard sorafenib treatment.

To explore the key factors regulating sorafenib sensitivity of HCC patients, we used publicly available dataset (GSE109211)^[^ ^23^ ^]^ to compare the mRNA expression profiles of tumor samples of sorafenib‐sensitive (*n* = 21) and ‐insensitive (*n* = 46) HCC patients (**Figure** **1**A). In order to identify the key factors, we analyzed the differentially expressed genes (DEGs) between these two groups of patients (Figure 1A) and the obtained 300 genes upregulated in the tumor samples of sorafenib‐insensitive patients were applied for gene ontology (GO) analysis. The results show that the DEGs are mainly enriched in “negative regulation of apoptotic process” of biological processes (BP, Figure S1A, Supporting Information), “extracellular exosome” of cellular components (CC, Figure S1B, Supporting Information), and “protein binding” of molecular function (MF, Figure S1C, Supporting Information). The gene information in these three GO entries were further intersected to obtain seven candidate genes (Figure 1B and Table S1, Supporting Information), and their interaction network was analyzed by using STRING database (Figure 1C). Among these candidate genes, CFL1 is particularly noted as it acts as a hub gene to interact with other six candidate genes and its expression level is negatively correlated with the expression of 30 sorafenib target pathway‐associated DEGs enriched in the GSE109211 dataset (Table S2 and Figure S2, Supporting Information), implying that CFL1 may play an important role in regulating sorafenib sensitivity of HCC patients.

To evaluate the clinical significance of CFL1 in HCC patients, we compared CFL1 expression in HCC tissues (*n* = 424) and normal liver tissues (*n* = 141) using TCGA (The Cancer Genome Atlas) database (Figure 1D). Indeed, CFL1 is highly expressed in HCC tissues compared to normal liver tissues, and high CFL1 expression indicates poor overall survival (OS, Figure 1E) and disease free survival (DFS, Figure 1F) of HCC patients. In addition, CFL1 expression is also positively correlated with the expression of proliferating cell nuclear antigen (Figure S3A, Supporting Information) and the marker of proliferation (Ki‐67, Figure S3B, Supporting Information). Besides analyzing the public database, we also used immunohistochemistry (IHC) to examine CFL1 expression in the surgically resected tumor samples of HCC patients (*n* = 120) (Figure 1G and Table S3, Supporting Information). Similar as the tendency in TCGA database, HCC patients with high CFL1 expression show poor OS (Figure 1H) and DFS (Figure 1I) compared to the patients with low CFL1 expression. Moreover, much higher CFL1 expression could be observed in the tumor tissues of HCC patients with relapse within postoperative 1 year (Figure 1J). To further evaluate the correlation between CFL1 expression and sorafenib sensitivity, we analyzed the survival benefit of HCC patients (*n* = 29) received standard sorafenib treatment in TCGA database. It can be found that the patients with low CFL1 expression show a better survival benefit from sorafenib treatment (Figure 1K). All these results indicate that CFL1 expression is closely correlated with sorafenib sensitivity of HCC patients, and its high expression could predict the poor prognosis of HCC patients.

### 2.2 CFL1 Silencing Enhances the Sorafenib Sensitivity of HCC Cells

**Figure 2:**
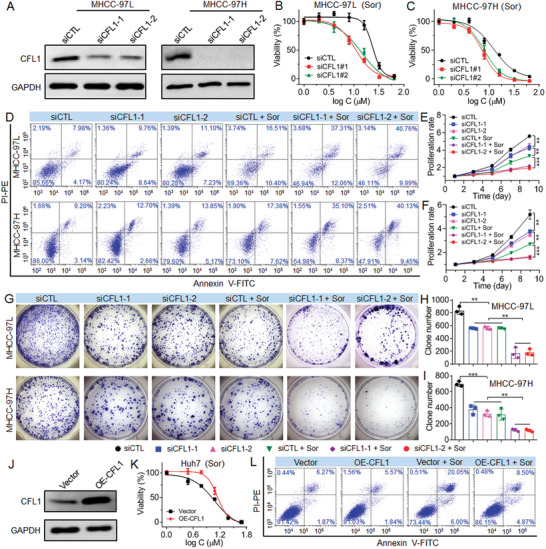
CFL1 silencing enhances the sorafenib sensitivity of HCC cells. A) Western blot analysis of CFL1 expression in MHCC‐97L and MHCC‐97H cells treated with 30 × 10^−9^ m siCFL1. B,C) Cytotoxicity of sorafenib against B) MHCC‐97L and C) MHCC‐97H cells treated with 30 × 10^−9^ m siCFL1. D) Flow cytometry analysis of the apoptosis of MHCC‐97L and MHCC‐97H cells treated with siCTL, siCFL1, siCTL followed by sorafenib (siCTL + Sor), or siCFL1 followed by sorafenib (siCFL1 + Sor) at an siRNA dose of 30 × 10^−9^ m and sorafenib dose of 8 × 10^−6^ m. E,F) Proliferation profiles of E) MHCC‐97L and F) MHCC‐97H cells treated with the formulas shown in (D). ** *p* < 0.01; *** *p* < 0.001. G–I) Clone formation and statistic results of MHCC‐97L and MHCC‐97H cells treated with the formulas shown in (D). J) Western blot analysis of CFL1 expression in Huh7 cells treated with 2 µg mL^−1^ CFL1 plasmid (OE‐CFL1). K) Cytotoxicity of sorafenib against Huh7 cells treated with 2 µg mL^−1^ CFL1 plasmid. L) Flow cytometry analysis of the apoptosis of Huh7 cells treated with blank plasmid (Vector), CFL1 plasmid, blank plasmid followed by sorafenib (Vector + Sor), or CFL1 plasmid followed by sorafenib (OE‐CFL1 + Sor) at a plasmid dose of 2 µg mL^−1^ and sorafenib dose of 5 × 10^−6^ m.

Having validated the important clinical significance of CFL1, we next explored how CFL1 regulates the sorafenib sensitivity of HCC. We first examined CFL1 expression in various types of HCC cells (MHCC‐97L, MHCC‐97H, HepG2, HCC‐LM3, BEL‐7402, PLC5, and Huh7) and found the negative correlation between CFL1 expression and the half‐inhibitory concentration (IC_50_) of sorafenib against HCC cells (Figures S4 and S5, Supporting Information). Due to the relatively higher CFL1 expression in MHCC‐97L and MHCC‐97H cells, they were chosen to explore how CFL1 regulates the sorafenib sensitivity. Moreover, because MHCC‐97L cells show much higher IC_50_ value of sorafenib (≈23.7 × 10^−6^ m) than other types of HCC cells, we used the one third of this IC_50_ value (≈8 × 10^−6^ m) to investigate the influence of CFL1 silencing on the sorafenib sensitivity of HCC cells. As shown in **Figure** **2**, when silencing CFL1 expression by siCFL1 (Figure 2A and Figure S6, Supporting Information), both MHCC‐97L and MHCC‐97H cells show an improved sorafenib sensitivity (Figure 2B,C). The IC_50_ value of sorafenib against MHCC‐97L cells decreases from ≈23.7 × 10^−6^ to 13.1 × 10^−6^ m while that of sorafenib against MHCC‐97H cells decreases from ≈12.3 × 10^−6^ to 7.7 × 10^−6^ m (Table S4, Supporting Information). The results of apoptosis analysis also demonstrate the ability of CFL1 to regulate sorafenib sensitivity (Figure 2D). CFL1 silencing could significantly enhance the sorafenib‐induced apoptosis of both MHCC‐97L and MHCC‐97H cells. The percentage of apoptotic cells reaches ≈50% after CFL1 silencing followed by sorafenib treatment (Figure S7, Supporting Information), which is around two fold higher than that of the cells treated with siCTL followed by sorafenib. With this improved sorafenib sensitivity, both MHCC‐97L and MHCC‐97H cells show weakened ability to proliferate (Figure 2E,F) and form clones (Figure 2G–I). Besides using MHCC‐97L and MHCC‐97L cells,^[^ ^24^ ^]^ we further evaluated whether CFL1 silencing could enhance the sorafenib sensitivity of HepG2 cells with high CFL1 expression (Figure S4, Supporting Information). Similarly, silencing CFL1 expression in HepG2 cells (Figure S8A,B, Supporting Information) could also dramatically enhance their sorafenib sensitivity, as demonstrated by the decreased IC_50_ value from ≈16.4 × 10^−6^ to 8.6 × 10^−6^ m (Figure S8C and Table S4, Supporting Information), increased apoptosis (Figure S8D,E, Supporting Information), suppressed proliferation (Figure S8F, Supporting Information), and decreased clone number (Figure S8G,H, Supporting Information).

To further validate the important role of CFL1 in regulating sorafenib sensitivity, we chose Huh7 cells with low CFL1 expression (Figure S4, Supporting Information) and examined the influence of CFL1 upregulation on their sorafenib sensitivity. As shown in Figure 2J,K, CFL1 upregulation could significantly impair the sorafenib sensitivity of Huh7 cells and the IC_50_ of sorafenib increases from ≈11.9 × 10^−6^ to 16.1 × 10^−6^ m (Table S4, Supporting Information). The similar tendency could be found in the apoptosis, proliferation, and clone formation experiments, in which CFL1 upregulation could effectively rescue the sorafenib‐induced apoptosis (Figure 2L and Figure S9A, Supporting Information) and promote the proliferation and clone formation of Huh7 cells (Figure S9B,C, Supporting Information).

**Figure 3:**
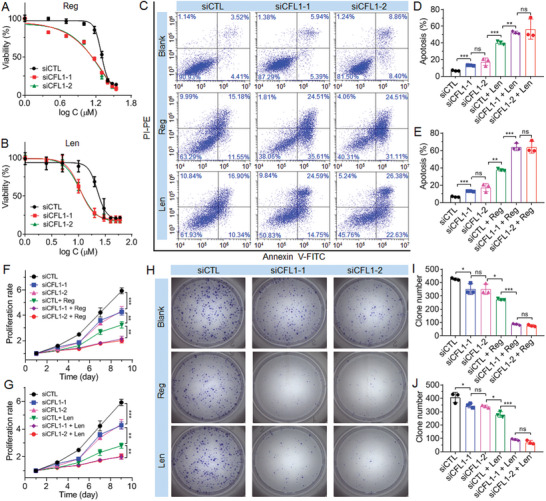
CFL1 silencing enhances the sensitivity of HCC cells to regorafenib and lenvatinib. A,B) Cytotoxicity of A) regorafenib and B) lenvatinib against MHCC‐97L cells treated with 30 × 10^−9^ m siCFL1. C) Flow cytometry analysis and D,E) statistic results of the apoptosis of MHCC‐97L cells treated with siCTL, siCFL1, siCTL followed by regorafenib (siCTL + Reg) or lenvatinib (siCTL + Leg), or siCFL1 followed by regorafenib (siCFL1 + Reg) or lenvatinib (siCFL1 + Leg) at an siRNA dose of 30 × 10^−9^ m and regorafenib dose of 8 × 10^−6^ m or lenvatinib dose of 12 × 10^−6^ m. *ns*, no significance; * *p* < 0.05; ** *p* < 0.01; *** *p* < 0.001. F,G) Proliferation profiles of MHCC‐97L cells treated with the formulas shown in (C). H) Clone formation and I,J) statistic results of MHCC‐97L cells treated with the formulas shown in (C).

Having confirmed the ability of CFL1 to regulate sorafenib sensitivity, we further evaluated whether CFL1 could also regulate the sensitivity of HCC cells to other clinically used TKIs as the benefits of these TKIs for advanced HCC patients are very limited.^[^ ^8^ ^]^ To this end, we down‐regulated CFL1 expression in MHCC‐97L cells and then, respectively, treated the cells with two clinically used TKIs, i.e., regorafenib and lenvatinib. As shown in **Figure** **3**, CFL1 silencing could significantly enhance the sensitivity of MHCC‐97L cells to both regorafenib and lenvatinib, as demonstrated by the decreased viability (Figure 3A,B), increased apoptosis (Figure 3C–E), suppressed proliferation (Figure 3F,G), and weakened clone formation ability (Figure 3H–J). All these results suggest that CFL1 could be a biomarker to predict the sensitivity of HCC cells to TKIs and silencing CFL1 expression could be a universal method to enhance the therapeutic outcomes of TKIs for advanced HCC.

### 2.3 CFL1 Silencing Inhibits PHGDH Transcription and Blocks Serine Synthesis and Metabolism to Enhance Intracellular ROS Level and Sorafenib Sensitivity

**Figure 4:**
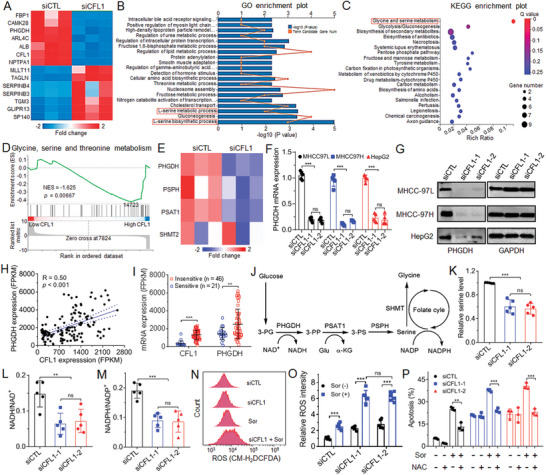
CFL1 silencing inhibits PHGDH transcription and blocks serine synthesis and metabolism to enhance the intracellular ROS level and sorafenib sensitivity of HCC cells. A) Heatmap of mRNA expression profiles of MHCC‐97L cells treated with 30 × 10^−9^ m siCFL1. B) GO, C) KEGG, and D) GSEA analysis of the DEGs in MHCC‐97L cells treated with 30 × 10^−9^ m siCFL1. E) Heatmap of the DEGs in the serine synthesis and metabolism pathway enriched in GO analysis shown in (B). F) mRNA and G) protein level of PHGDH in HCC cells after CFL1 silencing. H) Correlation between the expression level of CFL1 and PHGDH in GSE10921 dataset. I) Expression level of CFL1 and PHGDH in the tumor tissues of sorafenib‐sensitive (*n* = 21) and ‐insensitive patients (*n* = 46) in GSE109211 dataset. J) Schematic illustration of the serine synthesis and metabolism pathway. K–M) The level of K) serine, L) NADH/NAD^+^, and M) NADPH/NADP^+^ in MHCC‐97L cells treated with 30 × 10^−9^ m siCFL1. *ns*, no significance; ** *p* < 0.01; *** *p* < 0.001. N,O) Flow cytometry analysis of intracellular ROS level in MHCC‐97L cells treated with 30 × 10^−9^ m of siCFL1 and/or 8 × 10^−6^ m of sorafenib. P) Percentage of apoptosis of MHCC‐97L cells treated with 30 × 10^−9^ m siCFL1 followed by 8 × 10^−6^ m sorafenib in the presence or absence of 5 × 10^−3^ m NAC.

To explore the molecular mechanism how CFL1 regulates the sorafenib sensitivity of HCC, we examined the transcriptome change in MHCC‐97L cells after CFL1 silencing and the heatmap of DEGs is shown in **Figure** **4**A. The results of GO analysis (Figure 4B) and Kyoto Encyclopedia of Genes and Genomes (KEGG) analysis (Figure 4C) show that serine synthesis and metabolism is particularly enriched. In addition, the results of GeneSet Enrichment Analysis (GSEA) also indicate that serine metabolism is negatively regulated after CFL1 silencing (Figure 4D). Based on the results of DEGs in the serine synthesis and metabolism pathway enriched in GO analysis (Figure 4E), PHGDH, an important enzyme catalyzing serine synthesis,^[^ ^25^, ^26^ ^]^ is identified as the key factor that involves the CFL1‐regulated sorafenib sensitivity. To validate this result, we silenced CFL1 expression in multiple types of HCC cells and examined the change of PHGDH expression. Indeed, CFL1 silencing induces a significant decrease in PHGDH expression at both mRNA and protein level (Figure 4F,G), indicating that CFL1 could regulate PHGDH expression at the transcriptional level. In addition, we also examined the correlation between CFL1 and PHGDH using the publicly available dataset (GSE109211).^[^ ^23^ ^]^ The results show that CFL1 expression is positively correlated with PHGDH expression (Figure 4H), and both of them are highly expressed in the tumor tissues of sorafenib‐insensitive HCC patients compared to sorafenib‐sensitive HCC patients (Figure 4I).

It is known that PHGDH can catalyze 3‐phosphoglycerate (3‐PG) into 3‐phosphohydroxypyruvate (3‐PP), an intermediate for serine biosynthesis.^[^ ^25^ ^]^ In the process of serine synthesis and metabolism, two important antioxidants (i.e., nicotinamide adenine dinucleotide (NADH) and nicotinamide adenine dinucleotide phosphate (NADPH)) can be produced (Figure 4J), which can scavenge the excessive ROS and maintain intracellular redox balance.^[^ ^13^, ^15^, ^27^ ^]^ Since sorafenib can directly attack the ETC complexes and induce ROS production,^[^ ^12^ ^]^ we therefore speculate that high CFL1 expression could enhance PHGDH expression at the transcriptional level and thus promote serine synthesis and metabolism to produce high level of antioxidative NADH and NADPH, which could scavenge the excessive ROS triggered by sorafenib and thereby impair the sorafenib sensitivity of HCC cells. To validate this speculation, we examined the amount of serine in HCC cells using mass spectrometry (MS). As shown in Figure 4K, the level of serine decreases by ≈40% after silencing CFL1 expression in MHCC‐97L cells. With this decreased serine production, the ratio between antioxidants (NADH and NADPH) and their oxidized form (NAD^+^ and NADP^+^) dramatically decreases (Figure 4L,M), which could thus facilitate the accumulation of intracellular ROS. As shown in Figure 4N,O, CFL1 silencing or sorafenib treatment could moderately improve the intracellular ROS level in MHCC‐97L cells. However, the ROS level increases by around three fold when treating the cells with siCFL1 followed by sorafenib, thereby inducing much more apoptosis of MHCC‐97L cells (Figure 4P and Figure S8, Supporting Information). If adding the antioxidant *N*‐acetyl cysteine (NAC) or serine to rescue the ROS‐scavenging ability, the sorafenib sensitivity is significantly impaired, leading to decreased apoptosis of MHCC‐97L cells (Figure 4P and Figures S10 and S11, Supporting Information).

### 2.4 CFL1 Regulates PHGDH Transcription by Influencing Nrf2 Ubiquitination

**Figure 5:**
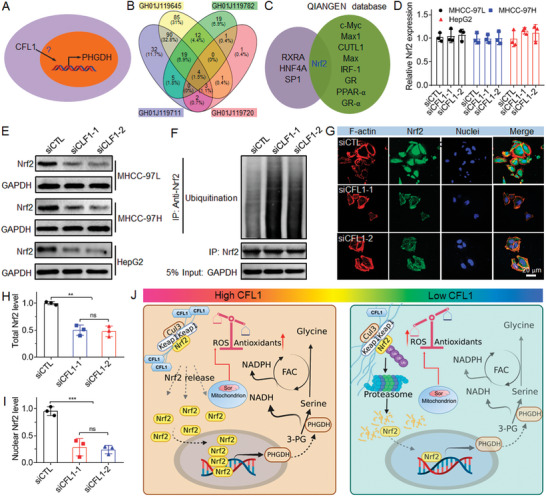
CFL1 regulates PHGDH transcription by influencing Nrf2 ubiquitination. A) Schematic illustration of the transcriptional regulation of PHGDH by CFL1. B) Prediction of the transcription factors binding to the promoter/enhancer regions of PHGDH gene. C) The intersection between four predicted transcription factors shown in (B) and another nine transcription factors in QIANGEN database. D) mRNA and E) protein level of Nrf2 in HCC cells treated with 30 × 10^−9^ m siCFL1. F) Nrf2 ubiquitination in MHCC‐97L cells treated with 30 × 10^−9^ m siCFL1. G) IF analysis of the expression of F‐actin and Nrf2 in MHCC‐97L cells treated with 30 × 10^−9^ m siCFL1. H) Total Nrf2 expression and I) its nuclear expression in MHCC‐97L cells treated with 30 × 10^−9^ m siCFL1. *ns*, no significance; ** *p* < 0.01; *** *p* < 0.001. J) Schematic illustration of the molecular mechanism of CFL1 in regulating sorafenib sensitivity. CFL1 could impair the Keap1‐Nrf2 interaction by depolymerizing F‐actin and thus promote the release and nuclear translocation of Nrf2 to enhance PHGDH transcription, which could thus enhance the serine synthesis and metabolism to accelerate the production of antioxidants for scavenging the excessive ROS triggered by sorafenib, ultimately leading to the weakened sorafenib sensitivity of HCC cells.

The above results indicate that CFL1 could regulate PHGDH expression at the transcriptional level, but the specific mechanism is unclear (**Figure** **5**A). In the public database (GENECARDS), many transcription factors can regulate PHGDH expression and four transcription factors are particularly noted, as each of them can bind to four different domains of the promoter/enhancer region of PHGDH gene (Figure 5B and Table S5, Supporting Information). To narrow the scope, we intersected these four transcription factors with another nine transcription factors that can bind the promoter/enhancer region of PHGDH gene in the QIANGEN database, and locked the only one transcription factor Nrf2 (Figure 5C), which has been recognized as the key regulator of intracellular redox homeostasis.^[^ ^28^ ^]^ This result is further demonstrated by the down‐regulated PHGDH expression after Nrf2 silencing (Figure S12, Supporting Information). With this information, we examined Nrf2 expression in HCC cells after CFL1 silencing, and the results show the mRNA level of Nrf2 does not change obviously (Figure 5D), but the protein level of Nrf2 is significantly down‐regulated in HCC cells (Figure 5E). These results suggest that CFL1 could regulate Nrf2 expression at a post‐transcriptional level such as ubiquitin‐proteasome degradation, as previous studies have clearly demonstrated that Nrf2 can be degraded via the classic ubiquitin‐proteasome pathway.^[^ ^29^ ^]^ To validate this speculation, we examined the level of Nrf2 ubiquitination after CFL1 silencing. As shown in Figure 5F, much higher level of Nrf2 ubiquitination could be observed after silencing CFL1 expression in MHCC‐97L cells.

We next explored how CFL1 regulates the ubiquitin‐proteasome degradation of Nrf2. It is known that Nrf2 can tightly bind a redox‐sensitive repressor Keap1,^[^ ^29^ ^]^ which acts as a scaffold that could not only bind a CUL3‐containing E3 ubiquitin ligase complex to induce Nrf2 ubiquitination, but also attach to F‐actin to enhance Nrf2 degradation by the proteasomes colocalized on F‐actin.^[^ ^30^, ^31^ ^]^ Recent studies have shown that disruption of F‐actin can weaken Keap1–Nrf2 interaction and enhance the entry of Nrf2 into the nucleus.^[^ ^7^, ^30^ ^]^ CFL1 is an essential actin‐regulating protein that can promote actin cytoskeleton reorganization by depolymerizing F‐actin into G‐actin,^[^ ^19^, ^20^ ^]^ which can thus augment the lamellipodium formation and extension to promote cancer progression.^[^ ^32^ ^]^ More importantly, the depolymerization of F‐actin into G‐actin could weaken the Keap1–Nrf2 interaction and may promote the nuclear translocation of Nrf2.^[^ ^30^ ^]^ To validate this statement, we observed the change of Nrf2 expression and intracellular location after CFL1 silencing. As shown in Figure 5G, Nrf2 shows high expression in the nucleus of MHCC‐97L cells and only a small amount of Nrf2 colocalizes with F‐actin in the cytoplasm (Figure 5H,I). After CFL1 silencing, both the ratio of G‐actin/F‐actin (Figure S13, Supporting Information) and Nrf2 expression are significantly decreased, and a majority of Nrf2 is positioned in the cytoplasm and colocalized with F‐actin (Figure 5G–I). All these results indicate that high CFL1 expression could enhance the nuclear translocation of Nrf2 via impairing the Keap1–Nrf2 interaction to promote PHGDH transcription, which could thus promote serine synthesis and metabolism to accelerate the production of antioxidative NADH and NADPH for scavenging the excessive ROS triggered by sorafenib, ultimately leading to the weakened sorafenib sensitivity of HCC cells (Figure 5J).

### 2.5 NPs‐Mediated CFL1 Silencing Enhances Sorafenib Sensitivity In Vitro

**Figure 6:**
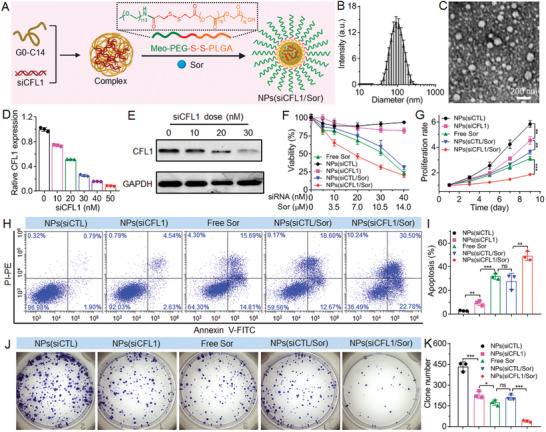
NPs‐mediated CFL1 silencing enhances the sorafenib sensitivity of HCC cells. A) Schematic illustration of the reduction‐responsive NPs made with the cationic lipid‐like compound G0‐C14 and amphiphilic copolymer Meo‐PEG‐S‐S‐PLGA. B) Size distribution and C) morphology of NPs(siCFL1/Sor) in aqueous solution. D) qRT‐PCR and E) western blot analysis of CFL1 expression in MHCC‐97L cells treated with the NPs(siCFL1/Sor) at different siCFL1 doses. F) Viability of MHCC‐97L cells treated with free sorafenib, NPs(siCTL), NPs(siCFL1), NPs(siCTL/Sor), or NPs(siCFL1/Sor) at different siCFL1 and/or sorafenib doses. G) Proliferation profile, H,I) apoptosis, and J,K) clone formation of MHCC‐97L cells treated with the formulas shown in (F) at an siRNA dose of 30 × 10^−9^ m and/or sorafenib dose of 10.5 × 10^−6^ m. *ns*, no significance; * *p* < 0.05; ** *p* < 0.01; *** *p* < 0.001.

Since CFL1 shows the strong ability to regulate sorafenib sensitivity, using NPs for systemic co‐delivery of sorafenib and siCFL1 could concurrently improve the accumulation of sorafenib in the HCC tumor tissues and enhance sorafenib sensitivity of HCC cells via silencing CFL1 expression. To this end, the NPs composed of a biodegradable methoxyl‐poly(ethylene glycol)‐*b*‐poly(lactic‐*co*‐glycolic acid) copolymer with a reduction‐responsive disulfide linker (denoted Meo‐PEG‐S‐S‐PLGA) and an amphiphilic cationic lipid‐like compound we previously developed (denoted G0‐C14, Figure S14, Supporting Information)^[^ ^33^ ^]^ were used to co‐encapsulate sorafenib and siCFL1 (**Figure** **6**A). This co‐delivery nanoplatform could not only respond to the highly concentrated glutathione (GSH) in tumor cells (2 × 10^−3^ to 10 × 10^−3^ m) to rapidly release loaded cargoes,^[^ ^34^, ^35^, ^36^ ^]^ but also facilitate to scavenge antioxidative GSH for improving the intracellular ROS level.^[^ ^37^ ^]^ As shown in Figure 6B,C, the NPs loading siCFL1 and sorafenib (denoted NPs(siCFL1/Sor)) show a spherical morphology with an averages size of ≈100 nm and encapsulation efficiency of siCFL1 and sorafenib of ≈80% and 40%, respectively. Due to the presence of disulfide bond in the Meo‐PEG‐S‐S‐PLGA copolymer, these NPs show a reduction‐responsive characteristic, as demonstrated by increased particle size, destroyed nanostructure, and fast drug release in the presence of GSH at a concentration (e.g., 10 × 10^−3^ m) closed to the level in tumor cells (Figure S15, Supporting Information).^[^ ^34^, ^35^ ^]^ More importantly, the NPs(siCFL1/Sor) could efficiently down‐regulate CFL1 expression by ≈80% in MHCC‐97L cells at a siCFL1 dose of 30 × 10^−9^ m (Figure 6D,E). With this efficient CFL1 silencing, the sorafenib sensitivity of MHCC‐97L cells is significantly improved and the cells thus show much lower viability (Figure 6F) and slower proliferation rate (Figure 6G) than that of cells treated with free sorafenib, NPs loading sorafenib and scrambled siRNA (denoted NPs(siCTL/Sor)) or NPs loading siCFL1 (denoted NPs(CFL1)). This improved sorafenib sensitivity could be also found in the results of apoptosis (Figure 6H,I) and clone formation experiments (Figure 6J,K), in which the NPs(siCFL1/Sor) show much stronger ability to induce apoptosis and inhibit clone formation of MHCC‐97L cells compared to free sorafenib, NPs(siCTL/Sor), and NPs(CFL1).

### 2.6 NPs‐Mediated CFL1 Silencing Enhances Sorafenib Sensitivity In Vivo

**Figure 7:**
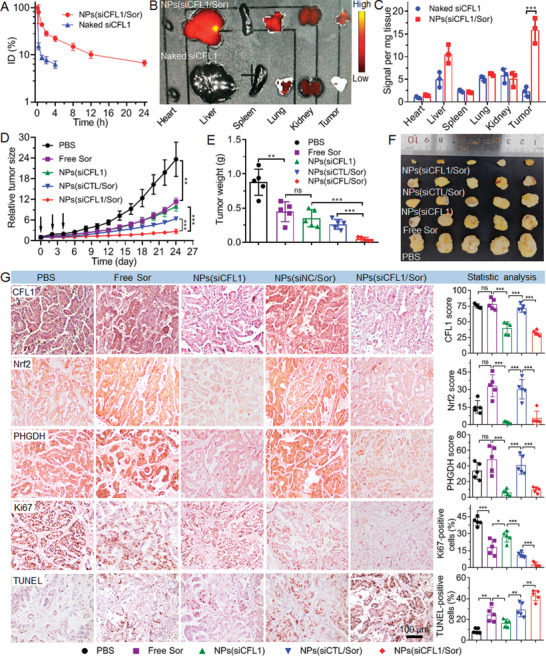
NPs‐mediated CFL1 silencing enhances the sorafenib sensitivity of HCC and inhibits HCC growth in MHCC‐97L xenograft tumor model. A) Blood circulation profile of naked siCFL1 and NPs(siCFL1/Sor). B) Overlaid fluorescence image of the tumors and main organs and C) biodistribution of naked siCFL1 and NPs(siCFL1/Sor) in the tumors and major organs of tumor‐bearing mice sacrificed at 24 h post injection. D) Tumor growth, E) tumor weight, and F) image of collected tumors of the tumor‐bearing mice treated with PBS, free sorafenib (Free Sor), NPs(siCFL1), NPs(siCTL/Sor), or NPs(siCFL1/Sor) at a 1 nmol siRNA dose per mouse and/or 6 mg kg^−1^ sorafenib dose per mouse. The intravenous injections are indicated by the arrows. *ns*, no significance; * *p* < 0.05; ** *p* < 0.01; *** *p* < 0.001. G) Expression of CFL1, Nrf2, PHGDH, Ki67, and TUNEL and statistical results determined by IHC staining analysis of the tumor tissues of mice after systemic treatment in each group.

Having demonstrated the efficient CFL1 silencing by NPs(siCFL1/Sor) to enhance sorafenib sensitivity in vitro, we next evaluated whether these NPs could use this ability to inhibit HCC tumor growth. The pharmacokinetics and biodistribution (BioD) of NPs(siCFL1/Sor) were first examined by intravenously injecting the NPs into healthy mice and MHCC‐97L xenograft tumor‐bearing mice (*n* = 3, 1 nmol siCFL1 dose per mouse), respectively. As shown in **Figure** **7**A, due to the protection by PEG chains,^[^ ^38^ ^]^ the NPs show a long blood circulation characteristic and around 10% of NPs(siCFL1/Sor) could be detected in the blood at 24 h post‐injection. With this long blood circulation, the NPs(siCFL1/Sor) show around seven fold higher accumulation in the tumor tissues compared to naked siCFL1 (Figure 7B,C). Moreover, most of these intratumoral NPs(siCFL1/Sor) could be internalized by tumor cells (Figure S16, Supporting Information). After confirming the high tumor accumulation of NPs(siCFL1/Sor), we finally evaluated their tumor inhibition ability via intravenous injection of these NPs into MHCC‐97L xenograft tumor‐bearing mice every 2 days (*n* = 5, 1 nmol siCFL1 dose per mouse). As expected, the administration of NPs(siCFL1/Sor) could significantly inhibit the tumor growth (Figure 7D–F) and there is less than three fold increase in the tumor size within an evaluation period of 24 days (Figure S17, Supporting Information). In comparison, the tumor size increases by more than six fold for the mice treated with free sorafenib, NPs(siCTL/Sor) or NPs(CFL1). The results of histological analysis of tumor tissues also indicate that the NPs(siCFL1/Sor) are the most effective in inhibiting tumor growth, as demonstrated by lower expression of CFL1, Nrf2, and PHGDH, more apoptosis (TUNEL), and less proliferation (Ki67) compared to the mice treated with other formulas (Figure 7G).

**Figure 8:**
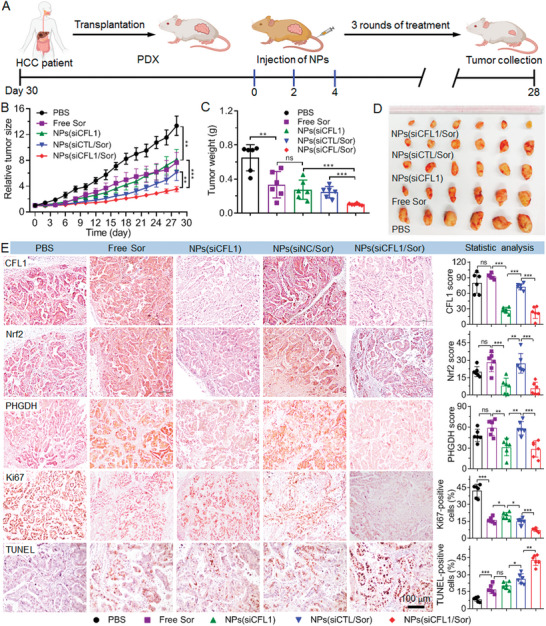
NPs‐mediated CFL1 silencing enhances the sorafenib sensitivity of HCC and inhibits HCC growth in PDX tumor model. A) Schematic illustration of PDX tumor inoculation and treatment of PDX tumor‐bearing mice. 30 days after tumor inoculation, tumor‐bearing mice were treated with PBS, free sorafenib (Free Sor), NPs(siCFL1), NPs(siCTL/Sor), or NPs(siCFL1/Sor) at a 1 nmol siRNA dose per mouse and/or 6 mg kg^−1^ sorafenib dose per mouse. B) Tumor growth, C) tumor weight, and D) image of the collected tumors of tumor‐bearing mice treated with the formulas in (A). *ns*, no significance; * *p* < 0.05; ** *p* < 0.01; *** *p* < 0.001. E) Expression of CFL1, Nrf2, PHGDH, Ki67, and TUNEL and statistical results determined by IHC staining analysis of the tumor tissues of mice after systemic treatment in each group.

To further evaluate the therapeutic effect of NPs(siCFL1/Sor), we collected surgically resected tumor tissues of HCC patients and established the patient‐derived xenografts (PDX) tumor‐bearing mouse model (**Figure** **8**A). As shown in Figure 8B–D, the intravenous administration of NPs(siCFL1/Sor) could also significantly inhibit the PDX tumor growth. The tumor size increases by around 3.5‐fold within an evaluation period of 28 days (Figure S18, Supporting Information), which is much lower than that of mice treated with free sorafenib, NPs(siCTL/Sor), or NPs(CFL1). Similar as the results of MHCC‐97L xenograft tumors, histological analysis also demonstrates that the NPs(siCFL1/Sor) are the most effective in suppressing CFL1 expression and inhibiting the PDX tumor growth (Figure 8E). Notably, the administration of NPs(siCFL1/Sor) does not influence the mouse weight (Figure S19, Supporting Information) and no apparent histological change could be observed in the tissues of major organs (Figure S20, Supporting Information), implying the low in vivo toxicity of NPs(siCFL1/Sor). This result is further verified by blood routine analysis (Figure S21, Supporting Information), in which the main parameters of liver and kidney function are in the normal range.

## 3 Discussion

At present, TKIs are still the first‐line treatment for advanced and unresectable HCC, but the low response rate of HCC patient has become a clinical pain‐point. The complex etiology and different pathological conditions of HCC patients significantly increase the difficulty to explore the intrinsic reasons. Prospective randomized controlled trial clinical studies have been recognized as a standard strategy to evaluate drug efficacy, in which pathology data of clinical patients and their comprehensive in‐depth sequencing data are particularly useful for exploring the key factors contributing to drug sensitivity. In this work, we used the publicly available dataset (GSE109211)^[^ ^23^ ^]^ and analyzed the mRNA sequencing data of sorafenib‐sensitive and ‐insensitive patients. Through comparing their DEGs, we identified that CFL1 may be the key factor affecting the therapeutic outcomes of sorafenib. Subsequent experiments further confirm that CFL1 could indeed regulate the sensitivity of HCC cells to both sorafenib and other clinically used TKIs (e.g., regorafenib and lenvatinib). As a classic actin‐binding protein, CFL1 primarily involves the remodeling of cytoskeleton and cell motility.^[^ ^19^, ^32^ ^]^ To the best of our knowledge, there is not any report about the function of CFL1 in regulating drug sensitivity. Our study first demonstrates that CFL1 could impair the Keap1–Nrf2 interaction via promoting the depolymerization of F‐actin into G‐actin and improve the nuclear translocation of Nrf2 to enhance PHGDH transcription and promote serine synthesis and metabolism, which could induce the production of more antioxidants to scavenge the excessive ROS triggered by sorafenib and thus impair sorafenib sensitivity of HCC cells. This regulatory mechanism is consistent with a recent study that the Nrf2‐mediated up‐regulation of PHGDH expression could weaken the sensitivity of HCC cells to sorafenib and other sorafenib‐like small molecule drugs.^[^ ^15^ ^]^ In fact, Nrf2 has been widely recognized as an important regulator of intracellular redox homeostasis. In the resting state, Nrf2 tightly binds to Keap1, a scaffold protein that can concurrently bind ubiquitin ligase and F‐actin to meditate the ubiquitin‐proteasome degradation of Nrf2.^[^ ^30^, ^31^ ^]^ Numerous previous studies have revealed that exogenous stimuli (e.g., ionizing radiation and chemotherapeutic drugs) can impair the Keap1–Nrf2 interaction and enhance the nuclear translation of Nrf2 to activate multiple pathways to maintain the intracellular redox homeostasis.^[^ ^39^ ^]^ The results in our work indicate that depolymerization of F‐actin by CFL1 could be another way to impair the Keap1–Nrf2 interaction and enhance the nuclear translocation of Nrf2 for the regulation of intracellular redox homeostasis.

In clinic, frequent administration of TKIs usually induces serious side effects including nausea and vomiting, diarrhea, skin rash, and musculoskeletal complaints.^[^ ^7^, ^21^ ^]^ In the past few decades, NPs‐mediated drug delivery has shown the advantages of increasing drug therapeutic efficacy and reducing toxic and side effects.^[^ ^3^, ^36^, ^40^ ^]^ We had previously developed various polymeric NPs especially the stimuli‐responsive NPs for drug delivery and cancer therapy.^[^ ^35^, ^41^ ^]^ These NPs show the characteristics of responding to biological stimuli (e.g., pH, reduction, hypoxia, and ROS) to achieve efficient drug delivery and improved anticancer effect. In this work, considering the high concentration of GSH in tumor cells^[^ ^34^, ^35^ ^]^ and the lack of clinically used inhibitors for CFL1, we therefore used the reduction‐responsive NPs for systemic co‐delivery of sorafenib and siRNA targeting CFL1 and evaluated their clinical translation potential for HCC therapy. In recent years, siRNA‐based therapeutics has made a breakthrough and several therapeutics (e.g., Onpattro, Givlaari, and Oxlumo) have been approved for clinical application.^[^ ^42^ ^]^ Among them, Onpattro is the first Food and Drug Administration (FDA)‐approved siRNA drug for the treatment of hereditary transthyretin amyloidosis (hATTR). In addition, numerous siRNA‐based anticancer therapeutics are under clinical studies. For example, STP707 contains two siRNA sequences targeting TGF‐*β* and COX‐2, and is now under phase I clinical trial (NCT05037149), which is expected to concurrently suppress the expression of TGF‐*β* and COX‐2 and synergistically inhibit tumor growth.^[^ ^43^ ^]^ Our work reveals that the reduction‐responsive NPs‐mediated co‐delivery system could highly accumulate in the tumor tissues and significantly enhance the sorafenib sensitivity of HCC tumors via silencing CFL1 expression, thereby leading to a significant inhibition of tumor growth without apparent toxicity. These results suggest that NPs‐mediated co‐delivery of sorafenib and siCFL1 could be a new therapeutic approach for the treatment of advanced HCC.

## 4 Conclusion

In summary, we have identified and demonstrated the important function of CFL1 in regulating sorafenib sensitivity of HCC. Molecular mechanism study reveals that high CFL1 expression could enhance the nuclear translocation of Nrf2 via impairing the Keap1–Nrf2 interaction to promote PHGDH transcription and thus improve serine synthesis and metabolism, which could accelerate the production of antioxidants (i.e., NADH and NADPH) to scavenge the excessive ROS triggered by sorafenib, ultimately leading to the weakened sorafenib sensitivity of HCC cells. Systemic co‐delivery of sorafenib and siCFL1 by reduction‐responsive NPs could effectively silence CFL1 expression and enhance sorafenib sensitivity in vivo, thereby leading to a significant inhibition of HCC tumor growth. CFL1 could be used to predict the prognosis of HCC patients and the NPs‐mediated co‐delivery of sorafenib and siCFL1 could be an effective strategy for the treatment of advanced HCC.

## 5 Experimental Section

### Patients and Tissue Samples

Surgically resected tumor samples of HCC patients (*n* = 120) were collected from Department of Hepatobiliary Surgery, Sun Yat‐Sen Memorial Hospital. All tumor samples were collected with informed consent from the donors according to the International Ethical Guidelines for Biomedical Research Involving Human Subjects (CIOMS). The study was approved by the Institutional Review Board (IRB) of Sun Yat‐Sen Memorial Hospital.

### Online Database

RNA‐sequencing data of tumor samples of sorafenib‐sensitive (*n* = 21) and insensitive (*n* = 46) HCC patients were derived from the GEO dataset (GSE109211).^[^ ^23^ ^]^ GO and GSEA enrichment analysis were performed using Sangerbox tools (http://vip.sangerbox.com). The expression CFL1 and PHGDH and the prognosis of HCC patients in TCGA database were downloaded from UCSC Xena (http://xena.ucsc.edu). Transcription factor prediction and verification data were obtained from the public database (https://www.genecards.org).

### Cell Culture

Human HCC cells (MHCC‐97L, MHCC‐97H, HepG2, Huh‐7, PLC5, BEL‐7402, and HCC‐LM3) were incubated in culture medium supplemented with 10% fetal bovine serum (FBS) and cultured at 37 °C in a humidified atmosphere containing 5% CO_2_.

### CFL1 Silencing and Over‐Expression

Human HCC cells (MHCC‐97L, MHCC‐97H, and HepG2) were seeded in 6‐well plates (5 × 10^4^ per well) and incubated in 2 mL of culture medium containing 10% FBS. After 24 h incubation, the complexes of lipofectamine 3000 (Lipo3k) and siCFL1 were added at an siRNA dose of 30 × 10^−9^ m. 24 h later, the medium was replaced by fresh medium and the cells were further incubated for another 48 h. Thereafter, the cells were digested by trypsin and the total RNA was extracted using Trizol for quantitative reverse transcription polymerase chain reaction (qRT‐PCR) analysis. The total protein was also extracted using lysis buffer supplemented with protease inhibitor cocktail and phenylmethanesulfonyl fluoride (PMSF) for western blot analysis. To up‐regulate CFL1 expression, Huh7 cells were seeded in 6‐well plates (5 × 10^3^ per well) and incubated with the complexes of Lipo3k and CFL1‐expressing plasmid at a plasmid dose of 2 µg mL^−1^. After 24 h incubation, the medium was refreshed and the cells were further incubated for another 48 h. Thereafter, the cells were digested by trypsin, and the total RNA and protein were extracted for qRT‐PCR and western blot analysis of CFL1 expression.

### Transcriptome Sequencing and Data Analysis

MHCC‐97L cells were seeded in 6‐well plates (5 × 10^4^ per well) and CFL1 expression in the cells was silenced according to the method described above. Subsequently, the total RNA was extracted with Trizol (Invitrogen, USA) for RNA‐sequencing analysis (Beijing Genomics Institution, China). The DEGs and downstream enrichment analysis were performed using the company's online interaction analysis system.

### Detection of Intracellular Serine Level and Metabolites

MHCC‐97L cells were seeded in 6‐well plates (5 × 10^4^ per well) and CFL1 expression in the cells was silenced according to the method described above. Subsequently, the cells were trypsinized and collected by centrifugation (1500 rpm, 10 min). After washing the cells with phosphate‐buffered saline (PBS) and deionized water containing 5% mannitol, respectively, the mixture of methanol, acetonitrile, and water in a volume ratio of 2/2/1 was added. After repeated freezing in the liquid nitrogen and thawing at room temperature, the mixture was centrifuged at 4 °C for 30 min (12 000 rpm) and the supernatant was finally collected. The amount of serine in the supernatant was determined by MS. The ratio of NADPH/NADP^+^ and NADH/NAD^+^ in the supernatant was also examined using the commercially available kits according to the manufacturer's protocols.

### Detection of Intracellular ROS

MHCC‐97L cells were seeded in 6‐well plates (5 × 10^4^ per well) and siCFL1 expression in the cells weas silenced according to the method described above. Thereafter, the cells were incubated in the medium containing 8 × 10^−6^ m sorafenib or the mixture of sorafenib (8 × 10^−6^ m) and NAC (5 × 10^−3^ m). After 24 h incubation, the cells were washed with PBS and then further cultured with the medium containing 2 × 10^−6^ m ROS probe (CM‐H2DCFDA, ThermoFisher) for 15 min. Subsequently, the cells were trypsinized and finally collected for ROS detection using a BD FACSAria III Flow Cytometry Analyzer.

### Preparation of NPs

The nanoprecipitation method was used to prepare the NPs.^[^ ^44^ ^]^ Briefly, 10 µL of siCFL1 solution (0.1 nmol/µL in water) was mixed with 50 µL of G0‐C14 solution (20 mg mL^−1^ in dimethylformamide), which was then added to the mixture of 15 µL of sorafenib solution (20 mg mL^−1^ in dimethyl sulfoxide) and 200 µL of Meo‐PEG‐S‐S‐PLGA copolymer solution (20 mg mL^−1^ in dimethylformamide). Subsequently, the mixture was added dropwise into 5 mL of deionized water under vigorous stirring (1000 rpm). The formed NPs (denoted NPs(siCFL1/Sor)) were purified using an EMD Millipore ultrafiltration device (MWCO 100 K) and finally dispersed in 1 mL of PBS solution. The NPs loading siCTL and sorafenib (denoted NPs(siCTL/Sor)) or only loading siCFL1 (denoted NPs(siCFL1)) or siCTL (denoted NPs(siCTL)) were also prepared according to the method described above.

### NPs‐Mediated CFL1 Silencing

MHCC‐97L cells were seeded in 6‐well plates (5 × 10^4^ per well) and incubated in 2 mL of medium containing 10% FBS for 24 h. Subsequently, the NPs(siCFL1/Sor) were added at different siRNA doses. After 24 h incubation, the cells were washed with PBS and further incubated in fresh medium for another 48 h. Thereafter, the cells were digested by trypsin and the total RNA was extracted using Trizol for qRT‐PCR analysis. The total protein was also extracted using lysis buffer supplemented with protease inhibitor cocktail and PMSF for western blot analysis.

### Animals

Healthy BALB/c (normal and nude) mice and NSG (NOD/SCID/IL2R*γ* ^null^) mice (male, 4–5 weeks old) were purchased from the Sun Yat‐Sen University Experimental Animal Center (Guangzhou, China). All in vivo studies were performed in accordance with a protocol approved by the Institutional Animal Care and Use Committee at Sun Yat‐Sen University (No. SYSU‐IACUC‐2022‐B0695).

### Establishment of HCC Tumor‐Bearing Mouse Models

MHCC‐97L xenograft tumor‐bearing mouse model was constructed by subcutaneous injection with 200 µL of MHCC‐97L cell suspension (a mixture of medium and Matrigel in 1:1 volume ratio) with a density 1 × 10^7^ cells mL^−1^ into the back region of healthy nude mice. When the tumor volume reached 100–150 mm^3^, the mice were used for the following in vivo experiments. For the establishment of PDX tumor‐bearing mouse model, fresh surgically resected tumor samples of HCC patients were cut into tiny pieces and then subcutaneously transplanted into back region of healthy NSG mice. When the tumor volume reached 100–150 mm^3^, the mice were used for the following in vivo experiments.

### Inhibition of HCC Tumor Growth

MHCC‐97L xenograft and PDX tumor‐bearing mice were, respectively, divided into five groups and given an intravenous injection of either i) PBS; ii) free sorafenib; iii) NPs(siCFL1); iv) NPs(siCTL/Sor); or NPs(siCFL1/Sor) once every 2 days at a siCFL1 dose of 1 nmol per mouse and/or sorafenib dose of 6 mg kg^−1^. All the mice were administrated by three consecutive injections and the tumor growth was monitored every 2 days by measuring perpendicular diameters using a caliper and tumor volume was calculated as: *V* = *W* ^2^ × *L*/2, where *W* and *L* are the shortest and longest diameters, respectively. After the systemic treatment, the mice were sacrificed at the end of evaluation period and the tumor tissues were collected. After fixing with 4% paraformaldehyde and then embedding in paraffin, the tumor tissues were sectioned for histological analysis.

### Statistical Analysis

Statistical results were presented as the mean ± S.D. of three or more independent experiments. Graphpad Prism (version 8.0) was used for illustrations, mapping, and statistical testing. Statistical significance was determined by a two‐tailed Student's *t*‐test. Kaplan–Meier curves and log‐rank tests were used to compare OS and DFS in different patient groups. A *p* value < 0.05 was considered statistically significant.

## Conflict of Interest

The authors declare no conflict of interest.

## Supporting information

Supporting Information

## Data Availability

The data that support the findings of this study are available in the supplementary material of this article.
