# Supplementary material for: Remodeling Serine Synthesis and Metabolism via Nanoparticles (NPs)‐Mediated CFL1 Silencing to Enhance the Sensitivity of Hepatocellular Carcinoma to Sorafenib
Source: Adv Sci (Weinh). 2023 May 18;10(19):2207118. doi: 10.1002/advs.202207118 (PMC10323624; doi:10.1002/advs.202207118)
Supplement: Supplementary file 1 — Supporting Information [file ADVS-10-2207118-s001.pdf]

ADVANCED SCIENCE Open Access

Supporting Information

for *Adv. Sci.*, DOI 10.1002/advs.202207118

Remodeling Serine Synthesis and Metabolism via Nanoparticles (NPs)-Mediated CFL1 Silencing to Enhance the Sensitivity of Hepatocellular Carcinoma to Sorafenib

Senlin Li, Lei Xu, Guo Wu, Ziqi Huang, Linzhuo Huang, Fengqian Zhang, Chunfang Wei, Qian Shen, Rong Li, Lei Zhang and Xiaoding Xu**

Supporting Information

for

Remodeling Serine Synthesis and Metabolism via Nanoparticles (NPs)-Mediated CFL1 Silencing to Enhance the Sensitivity of Hepatocellular Carcinoma to Sorafenib

Senlin Li,[a,b,c] *Lei Xu,*[a,b] *Guo Wu,*[a,b,d] *Ziqi Huang,*[a,b,c] *Linzhuo Huang,*[a,b] *Fengqian Zhang,*[a,b] *Chunfang Wei,*[a,b] *Qian Shen,*[a,b,d] *Rong Li,*[d] *Lei Zhang,*[a,b,c]* *and Xiaoding Xu*[a,b]*

[a] Guangdong Provincial Key Laboratory of Malignant Tumor Epigenetics and Gene Regulation, Guangdong-Hong Kong Joint Laboratory for RNA Medicine, Medical Research Center, Sun Yat-Sen Memorial Hospital, Sun Yat-Sen University, Guangzhou 510120, P. R. China

[b] Nanhai Translational Innovation Center of Precision Immunology, Sun Yat-Sen Memorial Hospital, Foshan 528200, P. R. China

[c] Department of Hepatobiliary Surgery, Sun Yat-Sen Memorial Hospital, Sun Yat-Sen University, Guangzhou 510120, P. R. China

[d] The Second Affiliated Hospital, Hengyang Medical School, University of South China, Hengyang 421001, P. R. China

*Corresponding author: xuxiaod5@mail.sysu.edu.cn; zhangl9@mail.sysu.edu.cn

1. Materials

Sorafenib was provided by APExBio and used directly. Dimethyl sulfoxide (DMSO), *N*-acetyl cysteine (NAC), reductive glutathione (GSH), serine, and *N*,*N*-dimethylformamide (DMF) were purchased from Sigma-Aldrich and used as received. Meo-PEG$_{5k}$-*S*-*S*-PLGA$_{11k}$ copolymer was provided by Xi'an Ruixi Biological Technology Company. Cationic lipid-like compound alkyl-modified polyamidoamine (PAMAM) dendrimer (G0-C14) was synthesized through ring opening of 1,2-epoxytetradecane by generation 0 of (PAMAM) dendrimer according to our previous studies [1, 2]. NADPH/NADP$^+$ (#ab65349) and NADH/NAD$^+$ (#ab65348) assay kits were purchased from Abcam. Click-iTTM TUNEL Colorimetric Detection Kit (#C10625) and ROS probe (CM-H2DCFDA, #C6827) were obtained from Thermo-Fisher. The siRNA targeting CFL1 and Ntf2 were acquired from IGE Bio. The siRNA sequences are as follows: siCFL1-1, 5'-AGC AUG AAU UGC AAG CAA A-3'; siCFL1-2, 5'-CUU CCA ACA CUA UGU UGA A-3'; siNRf2-1, 5'-GTC GTA TCC AGT GCA GGG TCC GAG G-3'; siNRf2-2, 5'-GGC CAG CTG TGA GTG TTT CTT-3'; siCTL, 5'-UUC UCC GAA CGU GUC ACG U-3'. Cy5-labled siCFL1-1 (denoted Cy5-siCFL1) was purchased from IGE Bio and Cy5 was labeled at the 5'-end of both sense and antisense strands. Dulbecco's Modified Eagle Medium (DMEM), penicillin-streptomycin, trypsin, and fetal bovine serum (FBS) were purchased from Invitrogen. All other reagents and solvents are of analytical grade and used without further purification.

2. Primers and antibodies

The primers for reverse transcription quantitative polymerase chain reaction (qRT-PCR) are as follows: CFL1, 5'-TAC GCC ACC TTT GTC AAG ATG-3' (forward sequence), 5'-CCT TGG AGC TGG CAT AAA TCA T-3' (reverse sequence); PHGDH, 5'-CTG CGG AAA GTG CTC

ATC AGT-3’ (forward sequence), 5’-TGG CAG AGC GAA CAA TAA GGC-3’ (reverse sequence); Nrf2, 5’-TCA GCG ACG GAA AGA GTA TGA-3’ (forward sequence), 5’-CCA CTG GTT TCT GAC TGG ATG T-3’ (reverse sequence); GAPDH, 5’-CAT CAT CTC TGC CCC CTC T-3’ (forward sequence), 5’-GGT GCT AAG CAG TTG GTG GT-3’ (reverse sequence). The detailed information of antibodies is as follows: CFL1 rabbit mAb (CST, #5175), PHGDH rabbit mAb (CST, #66350), Keap1 rabbit pAb (CST, #4678), ubiquitin rabbit pAb (CST, #3933), anti-rabbit IgG horseradish peroxidase (HRP)-linked secondary antibody (CST, #7074), GAPDH rabbit mAb (CST, #8884), Ki-67 mouse mAb (CST, #9449), Nrf2 mouse mAb (SANTA, sc-365949).

3. Screening and identification of sorafenib target pathway-associated differentially expressed genes (DEGs)

The sorafenib-sensitive (n = 21) and -insensitive (n = 46) HCC patients in GSE109211 dataset [3] were divided into a screening sample set and a validation sample set. The screening sample set included 10 sorafenib-sensitive HCC patients (GSM2935279, GSM2935280, GSM2935281, GSM2935282, GSM2935285, GSM2935288, GSM2935300, GSM2935310, GSM2935313, GSM2935333) and 10 sorafenib-insensitive HCC patients (GSM2935289, GSM2935292, GSM2935296, GSM2935297, GSM2935301, GSM2935302, GSM2935303, GSM2935304, GSM2935305, GSM2935307). Subsequently, the up-regulated DEGs (log FC > 1, $p < 0.01$) of sorafenib-sensitive HCC patients in the screening sample set were intersected with the sorafenib target pathway-associated genes from the Molecular Signatures Database (MSigDB). The obtained 36 genes shown in Table S2 were defined as the sorafenib target pathway-associated DEGs. The corresponding DEGs scores in the validation sample set, which contains 11

sorafenib-sensitive HCC patients and 36 sorafenib-insensitive HCC patients from the GSE109211 dataset, were calculated by using Gene Set Variation Analysis (GSVA) and their correlation with CFL1 expression level was finally analyzed.

4. Immunohistochemistry (IHC) and data analysis

IHC staining was performed on formalin-fixed paraffin-embedded tumor sections. Briefly, tumor slides were first heated to 60 ^{o}C for 1 h, desparaffinized with xylene (3 × 5 min), and washed with different concentrations of alcohol. After retrieval of antigen using DAKO target retrieval solution at 95-99 ^{o}C for 40 min, followed by washing, the slides were blocked with peroxidase blocking buffer (DAKO Company) for 5 min. After washing DAKO buffer the slides were incubated with the primary antibody (CFL1, PHGDH, Nrf2, and Ki67) diluted in DAKO antibody solution for 1 h. The slides were then washed and incubated with peroxidase-labeled polymer for 30 min. After washing and staining with DAB+ substrate-chromogen solution and hematoxylin, the slides we remounted and viewed under a MVX10 MacroView Dissecting scope equipped with OlympusDP80 camera. Image J software was employed to analyze CFL1 expression in the tumor tissues by combining the percentage of positively-stained tumor cells and staining intensity of positively-stained tumor cells. The staining intensity was graded as follows: 0, no staining; 1, weak staining (light yellow); 2, moderate staining (yellow-brown); 3, strong staining (brown). The percentage of cells at each staining intensity level was calculated and the CFL1 score was finally assigned using the following formula: [1 × (% cells 1+) + 2 × (% cells 2+) + 3 × (% cells 3+)]. The median value was used as the cut-off to define high CFL1 expression and low CFL1 expression in the tumor samples of HCC patients.

5. Reverse transcription quantitative polymerase chain reaction (qRT-PCR)

Total RNA was extracted from the cultured cells using Trizol and 1 μg of RNA was then reverse transcribed into cDNAs using a Superscript First-Strand cDNA Synthesis Kit (18080-051, Invitrogen, USA). qRT-PCR analysis was performed using SYBR Premix Ex Taq II kit (DRR081A, TAKARA, Japan) on a LightCycler 480 System (Roche, Switzerland).

6. Western blot

Equal amounts of proteins, as determined with a bicinchoninic acid (BCA) protein assay kit (Pierce/Thermo Scientific) according to the manufacturer's instruction, were added to sodium dodecyl sulfate polyacrylamide gel electrophoresis (SDS-PAGE) gels and separated by gel electrophoresis. After transferring the protein from gel to polyvinylidene difluoride (PVDF) membrane, the blot was blocked with 3% bovine serum albumin (BSA) in PBS solution containing 0.1% Tween 20 (PBST) for 1 h. Subsequently, the primary antibody (CFL1, PHGDH, and Nrf2) was added to incubate with the blot at 4 ^{o}C overnight. After washing the blot with PBST thrice, anti-rabbit IgG HRP-linked secondary antibody was added to incubate with the blot at 4 ^{o}C for 1 h. The protein expression was detected using an enhanced chemiluminescence detection system after washing the blot with PBST thrice.

7. Cytotoxicity assay

Human HCC cells (MHCC-97L, MHCC-97H, HepG2, and Huh7) were seeded in 96-well plates (5×10^3 per well) and incubated in 100 μL of culture medium containing 10% FBS. After 24 h incubation, CFL1 was down-regulated in MHCC-97L, MHCC-97H, and HepG2 cells using the complexes of Lipo3k/siCFL1 at a siRNA dose of 30 nM, while CFL1 was up-regulated in Huh7

cells using the complexes of Lipo3k/CFL1 plasmid at a plasmid dose of 2 μg/mL. Thereafter, the cells were further incubated in the medium containing sorafenib. After 24 h incubation, the cell viability was examined using AlamarBlue assay according to the manufacturer's protocol.

8. Detection of apoptosis

Human HCC cells (MHCC-97L, MHCC-97H, HepG2, and Huh7) were seeded in 6-well plates (5×10^4 per well) and incubated in 2 mL of culture medium containing 10% FBS. After 24 h incubation, CFL1 silencing or up-regulation was performed according to the method described above and the cells were further incubated with sorafenib (8 μM for MHCC-97L, MHCC-97H, and HepG2 cells and 5 μM for Huh7 cells). After 24 h incubation, the cells were digested by trypsin and collected by centrifugation (1500 rpm, 10 min). After staining with Annexin V and propidium iodide (PI), the apoptosis was detected using a BD FACSAria™ III Flow Cytometry Analyzer.

9. Proliferation assay

Human HCC cells (MHCC-97L, MHCC-97H, HepG2, and Huh7) were seeded in 6-well plates (3×10^4 per well) and CFL1 silencing or up-regulation was performed according to the method described above. Thereafter, the cells were incubated in the medium containing 8 μM sorafenib. After 24 h incubation, the cells were washed with phosphate buffered saline (PBS) solution and further incubated in fresh medium. At predetermined time points, the cell viability was measured by AlamarBlue assay according to the manufacturer's protocol. After each measurement, the AlamarBlue agent was replaced by fresh medium.

10. Clone formation assay

Human HCC cells (MHCC-97L, MHCC-97H, HepG2, and Huh7) were seeded in 6-well plates (3×10^4 per well) and CFL1 silencing or up-regulation was performed according to the method described above. Subsequently, the cells were further incubated with sorafenib (8 μM for MHCC-97L, MHCC-97H, and HepG2 cells and 5 μM for Huh7 cells). After 24 h incubation the cells were digested, seeded in 6-well plates (3000 cells per well), and further incubated in medium until cell clones could be observed. The clones were stained with crystal violet and imaged and counted under an Olympus optical microscope.

11. Ubiquitination assay

MHCC-97L cells were seeded in 6-well plates at a density of 50,000 cells/well and incubated in 2 mL of medium containing 10% FBS for 24 h. Subsequently, the cells were incubated Lipo3k/siCFL1 complexes at a siRNA dose of 30 nM for 24 h incubation. After washing with phosphate buffer saline (PBS) solution and further incubation in fresh medium for 48 h, MG132 was added to the medium at a dose of 20 nM. After 4 h incubation, the cells were digested by trypsin and the total protein was extracted using lysis buffer. Subsequently, the lysates were immunoprecipitated with the indicated antibodies on protein A/G beads (Life Technologies) overnight at 4 ^{o}C and the eluted proteins were detected by western blot.

12. Immunofluorescence (IF)

IF staining was performed on MHCC-97L cells after CFL1 silencing. Briefly, MHCC-97L cells were seeded in 6-well plates at a density of 50,000 cells/well and incubated in 2 mL of medium containing 10% FBS for 24 h. Subsequently, the cells were incubated Lipo3k/siCFL1 complexes

at a siRNA dose of 30 nM for 24 h incubation. After washing with PBS solution and further incubation in fresh medium for 48 h, the cells were fixed with paraformaldehyde (PFA) and then washed with ethanol and PBS thrice. After blocking with 10% FBS for 1.5 h, the slices were incubated with Nrf2 antibody at 4 ^{o}C for 1 h. After washing with PBS/0.2% triton X-100 thrice, Alexa Flour 488-conjugated secondary antibody (Goat anti-rat IgG, Abcam) was added for 1 h to stain the slices. Thereafter, the slices were washed with PBS thrice and then stained with Hoechst 33342. The images of the tumor vasculature were viewed on a ZEISS 800 CLSM.

13. Characterizations of NPs

The NPs loading siCFL1 and sorafenib (denoted NPs(siCFL1/Sor)) were prepared using the classic nanoprecipitation method [4] and finally suspended in mL of PBS solution. Size and zeta potential of the NPs were determined by dynamic light scattering (DLS, Malvern, USA). Their morphology was visualized on a Tecnai G^2 Spirit BioTWIN transmission electron microscope (TEM). Before observation, the sample was stained with 1% uranyl acetate and dried under air. To determine encapsulation efficiency (EE%) of siCFL1 and sorafenib, the NPs(Cy5-siCFL1/Sor) were also prepared using the nanoprecipitation method and suspended in 1 mL of PBS solution. A small volume (10 µL) of NP suspension was withdrawn and mixed with 20-fold DMSO. The fluorescence intensity of Cy5-siCFL1 was measured using a Synergy HT multi-mode microplate reader (BioTek, USA) and the EE% of siCFL1 was calculated according to the standard curve. Simultaneously, the mixture was analyzed by high-performance liquid chromatography (HPLC) to examine the amount of sorafenib and the EE% of siCFL1 was calculated according to the standard curve.

14. HPLC

HPLC analysis was performed on an Agilent Technologies 1200 Series System using C18 column (150 mm × 4.6 mm, 5 μm). The isocratic mobile phase consisted of acetonitrile and deionized water in the volume ratio of 65:35. Triethylamine (0.05%) and formic acid (0.05%) were added to the mobile phase to prevent peak tailing. The detector was set at 265 nm for data collection and analysis.

15. *In vitro* drug release

The NPs(Cy5-siCFL1/Sor) were suspended in 1 mL of PBS solution and then transferred to a Float-a-lyzer G2 dialysis device (MWCO 100 kDa, Spectrum) that was immersed in GSH-containing PBS solution at 37 ^{o}C. At a predetermined interval, 5 μL of the NP suspension was withdrawn and mixed with 20-fold DMSO. The fluorescence intensity of Cy5-siCFL1 was determined using a microplate reader and the amount of sorafenib was examined by HPLC.

16. Pharmacokinetics

Healthy male BALB/c normal mice were randomly divided into two groups (n = 3) and given an intravenous injection of either (i) free Cy5-siCFL1 or (ii) NPs(Cy5-siCFL1/Sor) at a siRNA dose of 1 nmol per mouse. At predetermined time intervals, orbital vein blood (20 µL) was withdrawn using a tube containing heparin, and the wound was pressed for several seconds to stop the bleeding. The fluorescence intensity of Cy5-siCFL1 in the blood was determined by a microplate reader.

17. Biodistribution

MHCC-97L xenograft tumor-bearing mice were randomly divided into two groups (n = 3) for intravenous injection of either (i) free Cy5-siCFL1 or (ii) NPs(Cy5-siCFL1/Sor) at a siRNA dose of 1 nmol per mouse. Twenty-four hours after the injection, the mice were imaged using an IVIS Lumina III (Perkin-Elmer, USA) imaging system. Organs and tumors were then harvested and imaged. To quantify the accumulation of Cy5-siCFL1 in tumors and organs, the fluorescence intensity of each tissue was quantified by Image-J.

18. Blood and histological analysis

Healthy male BALB/c mice were randomly divided into five groups (n = 3) and given an intravenous injection of either (i) PBS, (ii) free sorafenib, (iii) NPs(siCFL1), (iv) NPs(siCTL/Sor) or (v) NPs(siCFL1/Sor) at a siRNA dose of 1 nmol per mouse and/or sorafenib dose of 6 mg/kg. After three daily injections, the blood was collected 24 h post the final injection and serum isolated for measurements of representative blood parameters (AST, ALT, ALP, urea, creatinine, and total protein).

References

[1] Xu X, *et al.* Enhancing tumor cell response to chemotherapy through nanoparticle-mediated codelivery of siRNA and cisplatin prodrug. *Proc. Natl. Acad. Sci. U. S. A.* 2013, 110, 18638-18643.

[2] Xu X, *et al.* Redox-responsive nanoparticle-mediated systemic RNAi for effective cancer therapy. *Small 2*018, 14, e1802565.

[3] Pinyol R. *et al.* Molecular predictors of prevention of recurrence in HCC with sorafenib as adjuvant treatment and prognostic factors in the phase 3 STORM trial. *Gut* **2019**, *68*, 1065.

[4] Zhang L, *et al*. Self-Assembled lipid-polymer hybrid nanoparticles: a robust drug delivery platform. *ACS Nano* 2008, 2, 1696-1702.

Table S1. The relevant information of the 7 overlapped differential expression genes

ID	Gene	Expression in sorafenib-insensitive patients (FPKM)	Expression in sorafenib-sensitive patients (FPKM)	FC	-$\log_{10}$ (adj.P.Val)
ILMN_1705617	CFL1	1279.07	310.75	4.1	13.39
ILMN_1736567	CD74	1628.49	442.90	3.6	13.82
ILMN_1682763	ALB	3417.39	1067.29	3.2	13.39
ILMN_1711566	TIMP1	1794.63	511.01	3.5	13.39
ILMN_1782977	UBA52	6853.47	1554.54	4.4	13.04
ILMN_2094718	TPT1	3011.40	1041.15	2.8	7.99
ILMN_1684205	CIB1	4298.34	1066.40	9	13.39

Table S2. The relevant information of the 36 sorafenib target pathway-associated DEGs in the screening sample set

ID	Gene	Expression in sorafenib-insensitive patients (FPKM)	Expression in sorafenib-sensitive patients (FPKM)	FC	$-\log_{10}$ (adj.P.Val)
ILMN_2126802	RPS27L	288.7122	1650.532	6.171626	6.600326
ILMN_2409720	SLA2	302.4972	1407.214	4.999634	5.493495
ILMN_2233050	PLA2G2D	518.073	2263.08	4.247837	9.501689
ILMN_2297511	PODXL	1349.038	4677.274	4.106153	4.806875
ILMN_1667791	PPFIA4	346.624	1359.584	4.077422	3.280669
ILMN_3242004	ANXA8L1	327.8056	813.3606	3.885454	2.896196
ILMN_1701581	LAD1	416.2459	1410.438	3.623819	3.917215
ILMN_2326075	NTRK1	142.8956	487.386	3.502826	3.88941
ILMN_1675376	AURKC	181.253	453.026	3.326577	3.737549
ILMN_1690253	SYNPO2L	117.8619	418.9852	3.106401	3.68403
ILMN_1693749	BMP4	174.4994	422.9708	3.057137	3.090979
ILMN_1710590	PLA2G2E	161.1012	453.4771	3.025368	2.838632
ILMN_1719820	BDNF	144.6134	401.8194	3.006295	3.360514
ILMN_1786046	CASP9	346.9436	1012.837	2.996433	3.534617
ILMN_1663397	CAMK2B	208.6417	476.8206	2.859452	2.920819
ILMN_1757562	MPG	132.1787	384.8604	2.793678	3.193142
ILMN_1720849	PYGM	165.9769	481.3716	2.683502	3.417937
ILMN_1787212	CDKN1A	475.9836	1304.975	2.654037	3.120331

ILMN_1693060	VEGFA	839.2438	2036.954	2.602325	3.134304
ILMN_1678799	RAPGEF1	596.3441	1328.417	2.522355	2.853872
ILMN_1766275	PIK3CD	409.3251	967.6628	2.49755	2.826814
ILMN_1742764	UPP1	144.7623	358.12	2.472377	3.853872
ILMN_2334242	CREB1	247.3785	645.8696	2.394912	4.673664
ILMN_2411781	RYR1	179.1736	390.3449	2.346915	3.151811
ILMN_1781514	PCDH17	333.1562	800.5165	2.234511	2.798603
ILMN_2366967	WT1	136.573	303.2488	2.219788	3.241088
ILMN_1666445	CAMK2A	121.6942	261.6007	2.192945	2.939302
ILMN_1677564	TLX1	190.4615	449.6372	2.164613	2.832683
ILMN_1809813	PGF	290.1643	621.3502	2.143157	4.170053
ILMN_1803408	KRT18	161.896	369.1624	2.130682	3.853872
ILMN_1671547	HMGA2	124.5938	259.3546	2.110369	3.043351
ILMN_1709541	ATP2A1	141.377	317.3798	2.090361	2.982967
ILMN_2288976	PDE4B	114.087	243.7502	2.0894	3.179799
ILMN_1698739	UNC45B	103.3507	226.0379	2.088388	3.779892
ILMN_2199516	ZNF28	262.0283	506.9006	2.040761	3.17134
ILMN_1766363	FLT3	149.9849	335.6778	2.035983	2.785156

Table S3. The information of surgically resected tumor samples from 120 HCC patients

CFL1 expression			
Variable	High (60)	Low (60)	*p* value
Age			
≤50	31	30	1.00
>50	29	30	
Grade			
I+II	40	35	1.00
III	20	25	
T stage			
T1+T2	20	53	< 0.01
T3+T4	40	7	
N stage			
N0	54	55	1.00
N1	6	5	
Etiology			
HBV	53	41	0.99
Others	7	19	
Ki67 expression			
≤14%	3	17	0.89
>14%	57	43	

Table S4. Half inhibitory concentration (IC_{50}) of sorafenib against different HCC cells

Cell type	siCTL	siCFL1	Vector	OE-CFL1
MHCC-9L	23.7 μM	13.1 μM	/	/
MHCC-97H	12.3 μM	7.7 μM	/	/
HepG2	16.4 μM	8.6 μM	/	/
Huh7	/	/	11.9 μM	16.1 μM

Table S5. Transcription factors binding to four different domains in the promoter/enhancer region of PHGDH gene in GeneCards database

Domain	Transcription factors
GH01J119711	HCFC1 DPF2 ATF2 SIX5 JUND ZNF184 ZNF600 ZIC2 ZNF10 BRCA1 CEBPG YY1 HIC1 ZBTB10 CEBPA REST NFIC CEBPB MBD2 CHD2 ZNF592 NONO MNT HES1 TBP ARNT ZNF341 MAX ZNF217 EP300 IRF2 SMAD5 POLR2A ELF1 BHLHE40 ETS1 ZNF610 SAP130 CTBP1 ZFP64 SIN3A SP2 TARDBP GABPA THAP11 NKRF SMARCA5 ZBTB33 SIN3B SAFB RXRA ZNF316 SKIL ETV4 FOSL2 DRAP1 ZKSCAN1 NFE2 DMAP1 ZNF143 TEAD3 CBX1 ATF1 RAD21 ZNF843 HLTF SUPT5H ZBTB40 ZBTB48 KLF16 SPI1 ZNF318 PRDM6 ZBTB1 ZHX1 IRF9 CTCF MAFK FOXK2 ZNF24 EP400 ZNF189 MYC NRF1 ZEB2 MAFG INSM2 IKZF1 ZFP37 RB1 HNF4A EGR1 GATAD1 NR2F1 HMG20B SP1 TAF1 MTA3 HNRNPL ATF4 ZNF518A ZBTB21 BACH1 NFIB GLIS1 LARP7 TFAP4 ZNF398 TEAD4 MAFF ZFP69B GATA4 ZFX ELF3 CREM KLF1 SP7 PHF20 L3MBTL2 HDAC1 NFIA GABPB1 TRIM28 JUN ZNF366 ZNF561 ARID4B ARID1B ZNF785 NFATC3 SMARCE1 ZBTB7A KLF10 YY2 MLLT1 ZNF324 ARID3A HLF SOX13 ZSCAN21 TBL1XR1 ATF3 PHF21A KDM1A SP3 ZSCAN4 ZBTB8A DACH1 ZNF660 ZXDB EGR2 GTF2F1 XRCC5 NFE2L2 ZNF629 ZNF692 ZNF687 PATZ1 OSR2 LEF1 KAT8 RNF2 HDGF ZNF558 TRIM24 ZNF239 MZF1 PRDM10 ZBTB17 GLIS2 ZNF626 SOX6 NFRKB EED IKZF2 ELF4 PHF8 ATF7 GATAD2B CBX3 ZSCAN18 KLF8 POLR2B TCF7 TSHZ1 ZBTB20 GATAD2A ZNF121 FOS MEF2D HMBOX1 ZBTB11 RBFOX2 AFF1 NR2F2 TFE3 BRD9 ZNF423 ZFHX2 ZGPAT ZBTB2 PML SKI TEAD1 ZBTB26 HDAC2 SMARCA4 EMSY ZNF335 MIER1 ZBTB44 MGA ERF UBTF
GH01J119645	HCFC1 DPF2 ATF2 SIX5 JUND ZNF184 ZNF600 ZIC2 ZNF10 BRCA1 CEBPG YY1 HIC1 ZBTB10 CEBPA REST NFIC CEBPB MBD2 CHD2 ZNF592 NONO MNT HES1 TBP ARNT ZNF341 MAX ZNF217 EP300 IRF2 SMAD5 POLR2A ELF1 BHLHE40 ETS1 ZNF610 SAP130 CTBP1 ZFP64 SIN3A SP2 TARDBP GABPA THAP11 NKRF SMARCA5 ZBTB33 SIN3B SAFB RXRA ZNF316 SKIL ETV4 FOSL2 DRAP1 ZKSCAN1 NFE2 DMAP1 ZNF143 TEAD3 CBX1 ATF1 RAD21 ZNF843 HLTF SUPT5H ZBTB40 ZBTB48 KLF16 SPI1 ZNF318 PRDM6 ZBTB1 ZHX1 IRF9 CTCF MAFK FOXK2 ZNF24 EP400 ZNF189 MYC NRF1 ZEB2 MAFG INSM2 IKZF1 ZFP37 RB1 HNF4A EGR1 GATAD1 NR2F1 HMG20B SP1 TAF1 MTA3 HNRNPL ATF4 ZNF518A ZBTB21 BACH1 NFIB GLIS1 LARP7 TFAP4 ZNF398 TEAD4

	MAFF ZFP69B GATA4 ZFX ELF3 CREM KLF1 SP7 PHF20 L3MBTL2 HDAC1 NFIA GABPB1 TRIM28 JUN ZNF366 ZNF561 ARID4B ARID1B ZNF785 NFATC3 SMARCE1 ZBTB7A KLF10 YY2 MLLT1 ZNF324 ARID3A HLF SOX13 ZSCAN21 TBL1XR1 ATF3 PHF21A KDM1A SP3 ZSCAN4 ZBTB8A DACH1 ZNF660 ZXDB EGR2 GTF2F1 XRCC5 NFE2L2 ZNF629 ZNF692 ZNF687 PATZ1 OSR2 LEF1 KAT8 RNF2 HDGF ZNF558 TRIM24 ZNF239 MZF1 PRDM10 ZBTB17 GLIS2 ZNF626 SOX6 NFRKB EED IKZF2 ELF4 PHF8 ATF7 GATAD2B CBX3 ZSCAN18 KLF8 POLR2B TCF7 TSHZ1 ZBTB20 GATAD2A ZNF121 FOS MEF2D HMBOX1 ZBTB11 RBFOX2 AFF1 NR2F2 TFE3 BRD9 ZNF423 ZFHX2 ZGPAT ZBTB2 PML SKI TEAD1 ZBTB26 HDAC2 SMARCA4 EMSY ZNF335 MIER1 ZBTB44 MGA ERF UBTF
GH01J119782	CEBPG YY1 CEBPA CEBPB ZNF341 CUX1 CTBP1 NONO FOXA2 ZNF521 RXRA SMAD4 ZBTB33 RARA BCL6 JUND MAX IRF9 SOX5 MAFG SMARCE1 HNF4A HMG20A RAD21 DPF2 FOXA1 REST RXRB ZNF217 NR2F6 SP1 SP7 RFX5 ZNF366 BCL6B NFIL3 ZNF644 HLF ATF3 MNT PKNOX1 ZNF791 GATAD2A FOS ZNF629 MAFK U2AF2 ZNF512 RFX1 ESRRA PRDM10 HNF4G SOX13 IKZF2 ZNF512B FOXA3 ZBTB20 MTA2 NR2F2 TEAD1
GH01J119720	POLR2A RXRA RBM22 HNF4A ATF2 NR2F6 PPARG MIXL1 RBFOX2 NR2F2 TFE3 SP1

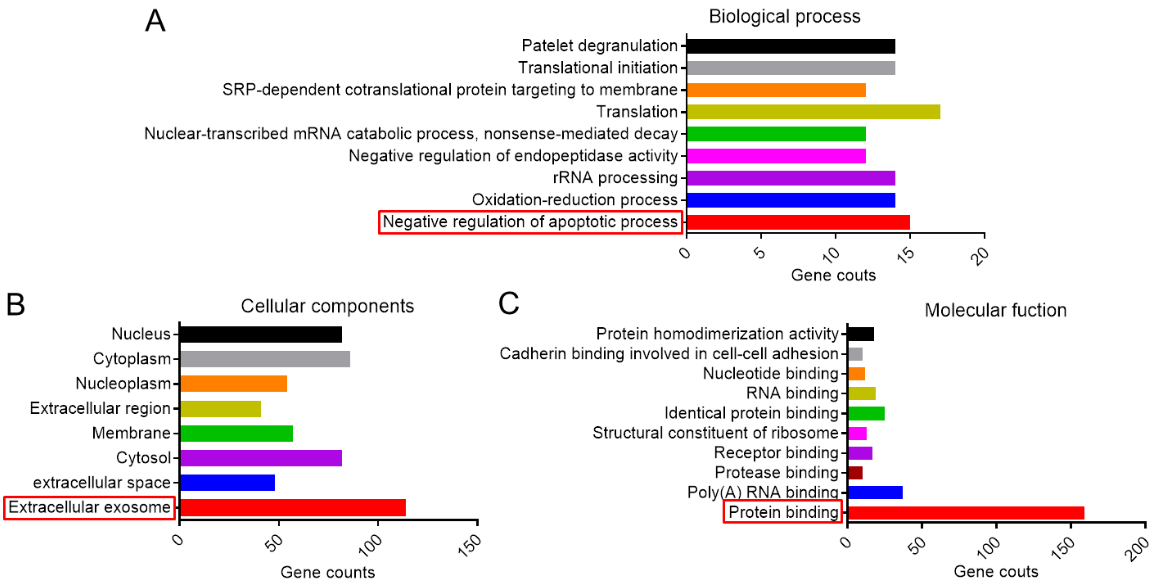

Figure S1. Gene Ontology (GO) analysis results of differentially expressed genes (DEGs) in the mRNA expression profiles of tumor tissues of sorafenib-sensitive (n = 21) and -insensitive HCC patients (n = 46) in GSE109211 dataset.

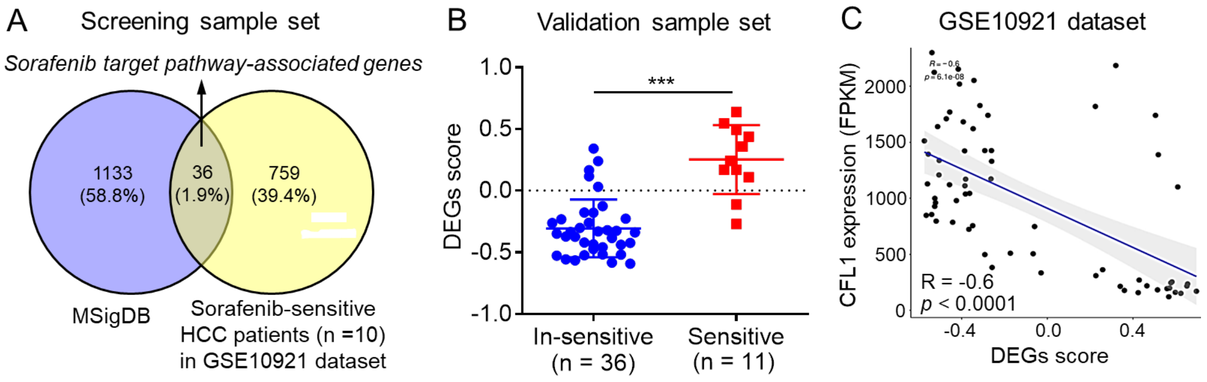

Figure S2. (A) Schematic illustration of the definition and selection of sorafenib target pathway-associated DEGs using the screening sample set. (B) The sorafenib target pathway-associated DEGs score in validation sample set, which contains 11 sorafenib-sensitive HCC patients and 36 sorafenib-insensitive HCC patients from the GSE109211 dataset. (C) The correlation between CFL1 expression and sorafenib target pathway-associated DEGs score in the tumor tissues of sorafenib-sensitive (n = 21) and -insensitive patients (n = 46) from the GSE10921 dataset.

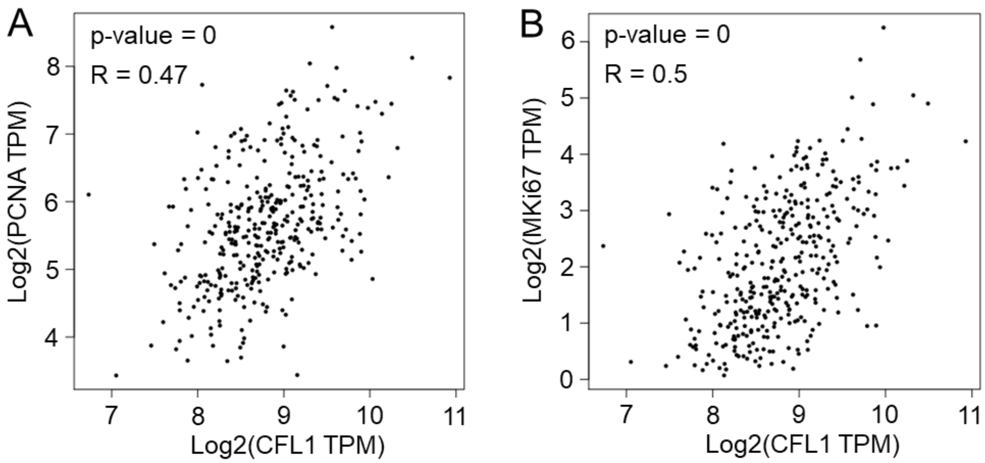

Figure S3. The correlation between CFL1 and proliferating cell nuclear antigen (PCNA) (A) or Ki-67 (B) in TCGA database.

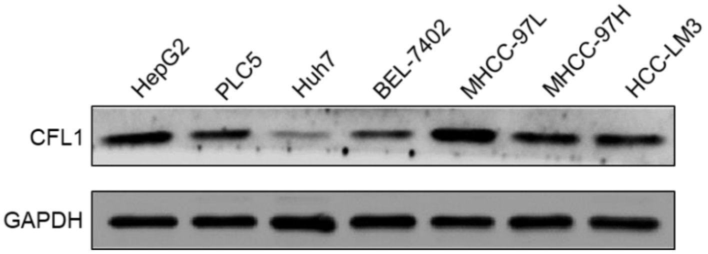

Figure S4. Western blot analysis of CFL1 expression in different human HCC cell lines.

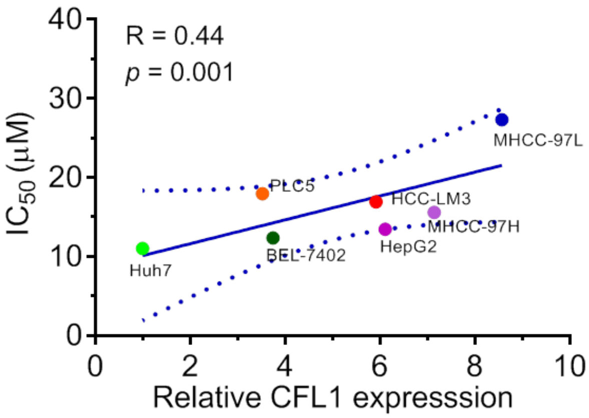

Figure S5. The correlation between the IC_{50} values of sorafenib against various HCC cell lines and their CFL1 expression level.

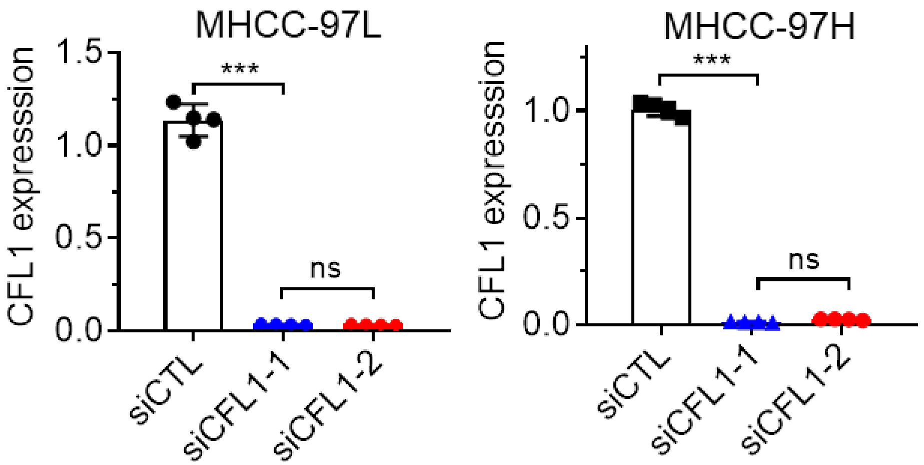

Figure S6. qRT-PCR analysis of CFL1 expression in MHCC-97L and MHCC-97H cells treated with siCFL1 at a siRNA dose of 30 nM.

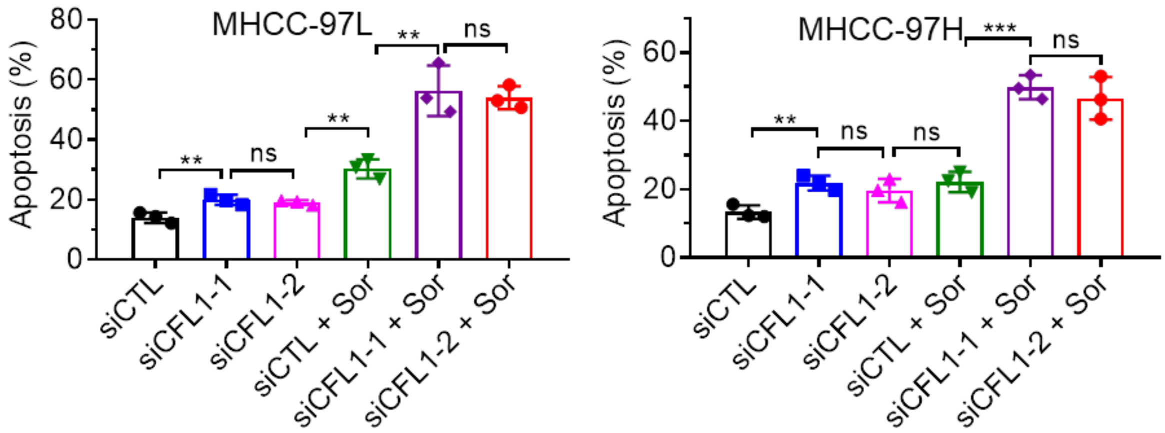

Figure S7. Percentage of apoptosis determined by flow cytometry analysis of MHCC-97L and MHCC-97H cells treated with siCTL, siCFL1, siCTL followed by sorafenib (siCTL + Sor), or siCFL1 followed by sorafenib (siCFL1 + Sor) at a siRNA dose of 30 nM and sorafenib dose of 8 μM. *ns*, no significance; ** $p < 0.01$; *** $p < 0.001$.

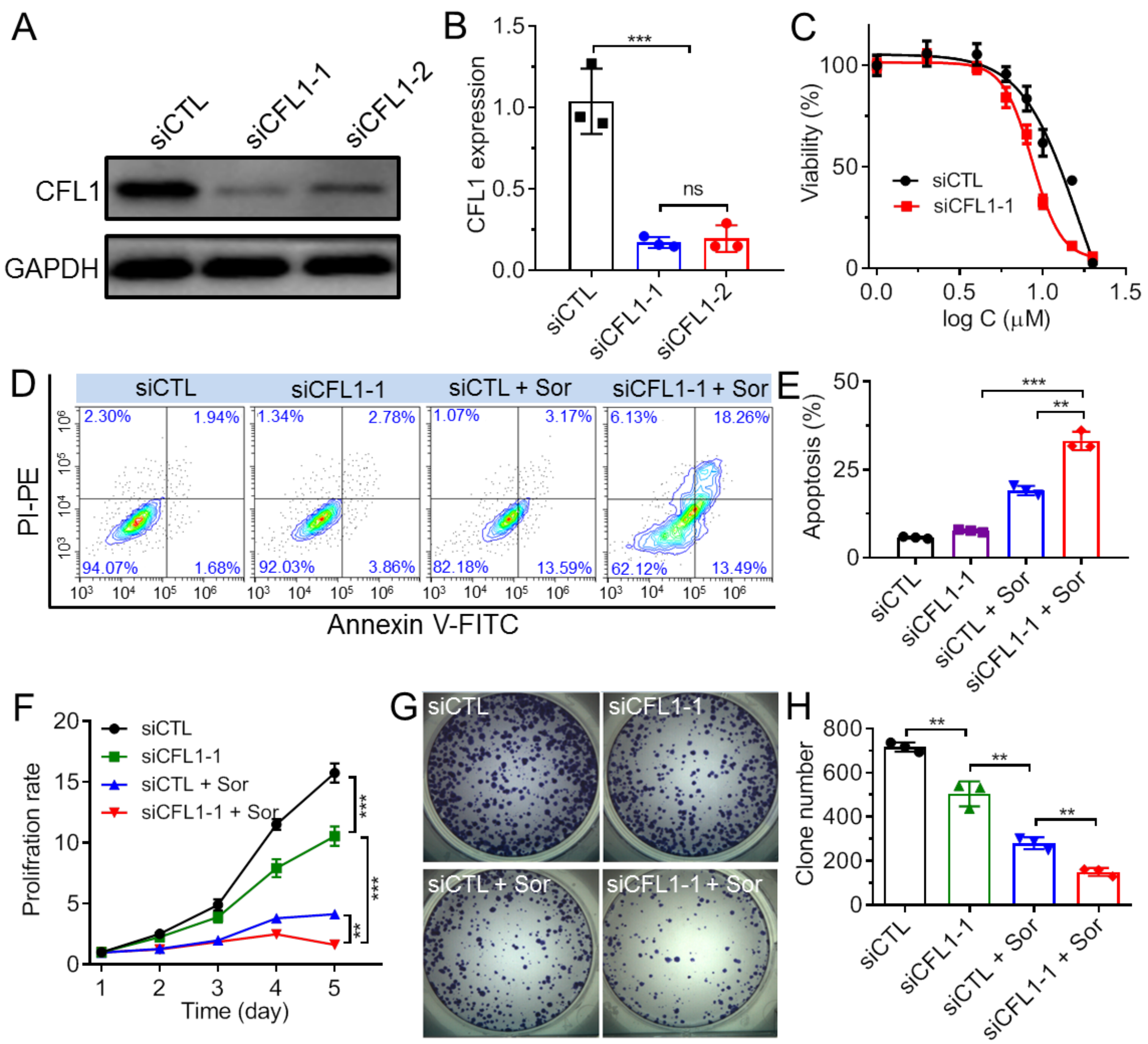

Figure S8. (A, B) Western blot (A) and qRT-PCR analysis of CFL1 expression in HepG2 cells treated with 30 nM siCFL1. (C) Viability of HepG2 cells treated with 30 nM siCFL1 and different concentrations of sorafenib. (D, E) Flow cytometry analysis (D) and statistic results (E) of the apoptosis of HepG2 cells treated with siCTL, siCFL1, siCTL followed by sorafenib (siCTL + Sor) or siCFL1 followed by sorafenib (siCFL1 + Sor) at a siRNA dose of 30 nM and sorafenib dose of 8 μM. ** $p < 0.01$; *** $p < 0.001$. (F) Proliferation profiles of HepG2 cells treated with the formulas shown in (D). (G, H) Clone formation (G) and statistic results (H) of HepG2 cells treated with the formulas in shown in (D).

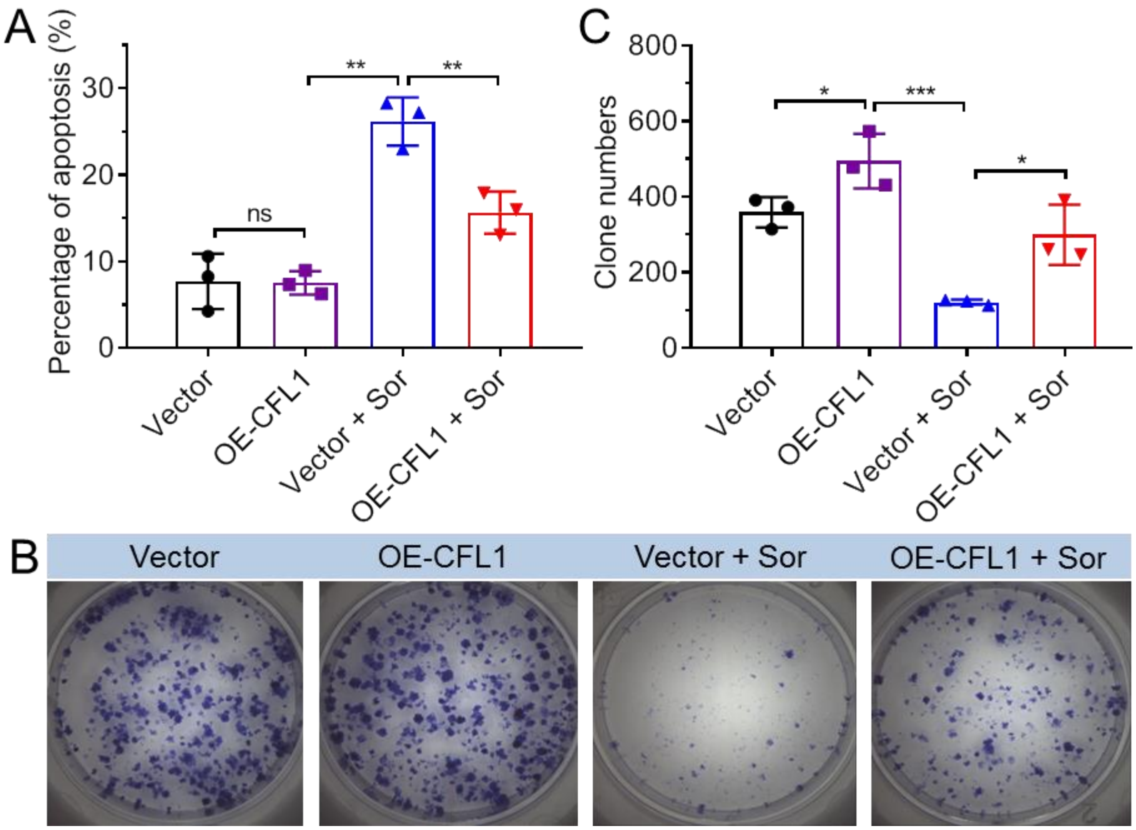

Figure S9. (A) Percentage of apoptosis determined by flow cytometry analysis of the apoptosis of Huh7 cells treated with blank plasmid (Vector), CFL1 plasmid, blank plasmid followed by sorafenib (Vector + Sor), or CFL1 plasmid followed by sorafenib (OE-CFL1 + Sor) at a plasmid dose of 2 μg/mL and sorafenib dose of 5 μM. (B, C) Clone formation (B) and statistic results (C) of Huh7 cells treated with the formulas shown in (A). *ns*, no significance; * $p < 0.05$; ** $p < 0.01$; *** $p < 0.001$.

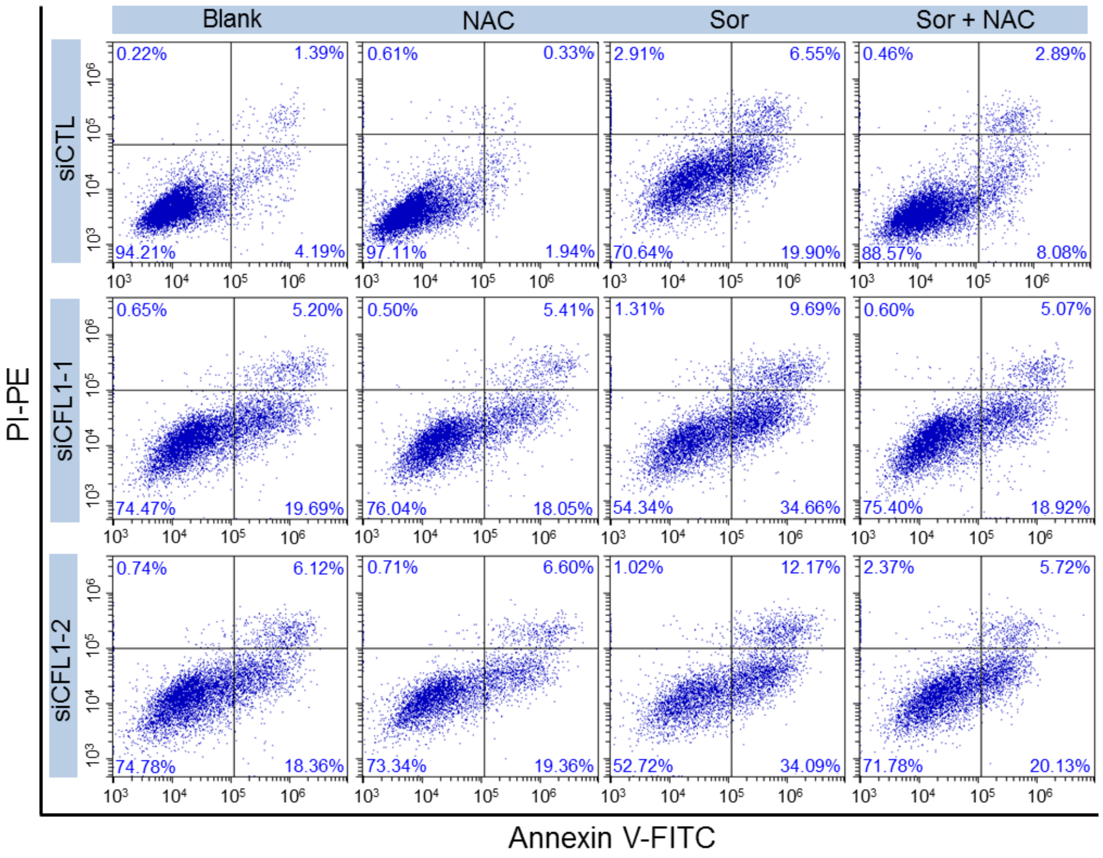

Figure S10. Flow cytometry analysis of the apoptosis of MHCC-97L cells treated with 30 nM siCTL or siCFL1 followed by 8 μM sorafenib in the presence or absence of 5 mM NAC.

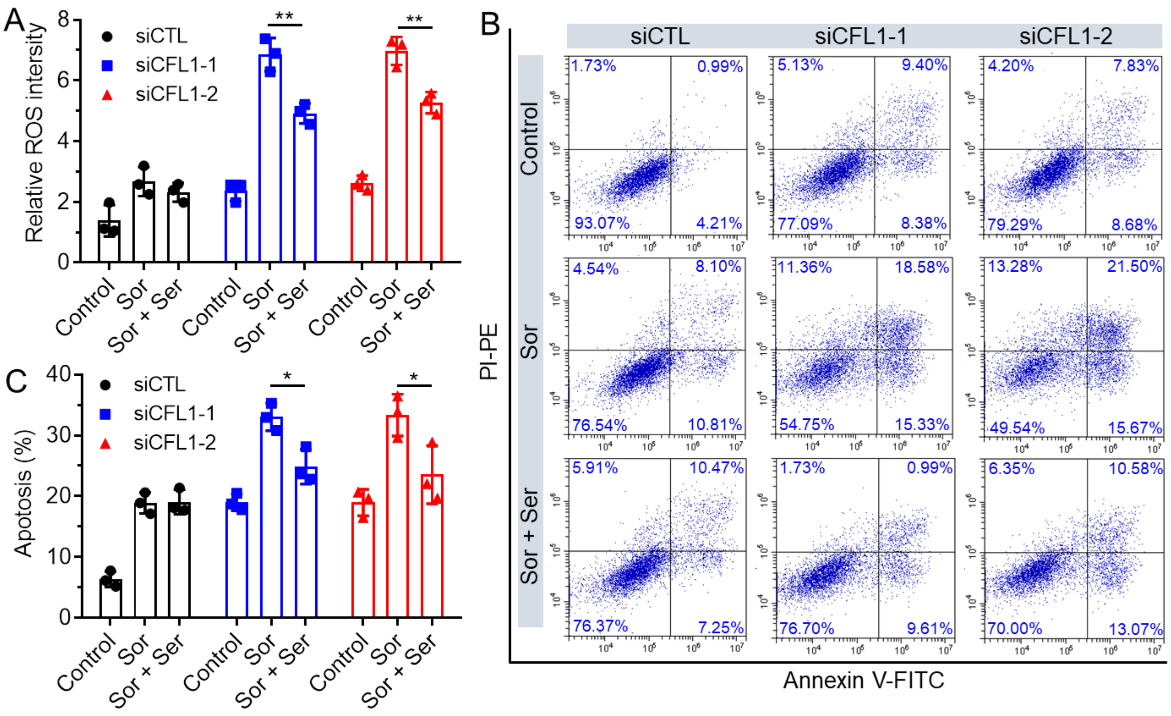

Figure S11. (A) Relative ROS level determined by flow cytometry analysis of MHCC-97L cells treated with 30 nM of siCFL1 followed by 8 μM of sorafenib and/or 100 mM serine. (B, C) Flow cytometry analysis (B) and statistic results (C) of the apoptosis of MHCC-97L cells treated with the formulas shown in (A). * $p < 0.05$; ** $p < 0.01$.

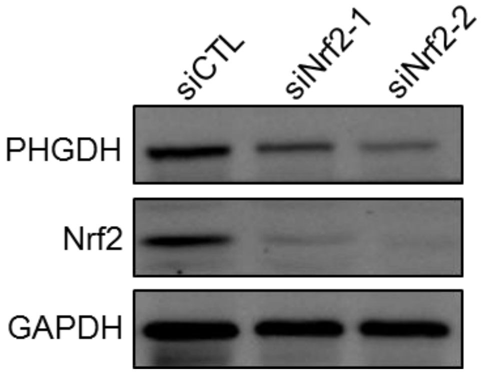

Figure S12. Western blot analysis of PHGDH expression in MHCC 97L cells treated with 30 nM Nrf2 siRNA (siNrf2).

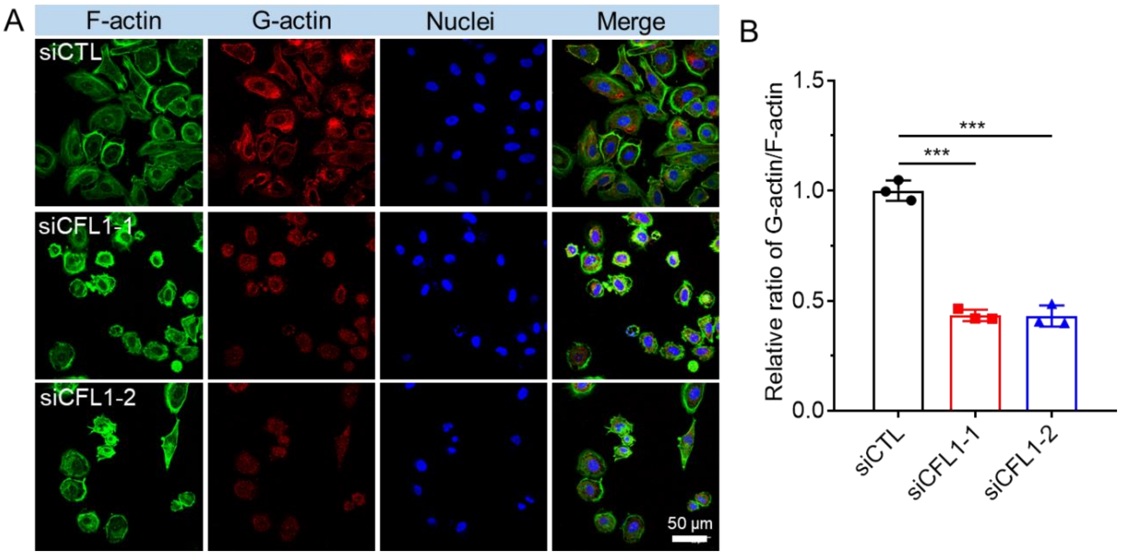

Figure S13. (A) IF analysis of F-actin and G-actin in MHCC-97L cells treated with siCFL1 at a siRNA dose of 30 nM. F-actin and G-actin were stained with FITC-phalloidin and Alexa Fluor 594-conjugated deoxyribonuclease I, respectively. (B) Relative ratio of G-actin/F-actin determined from IF analysis of MHCC-97L cells shown in (A). *** $p < 0.001$.

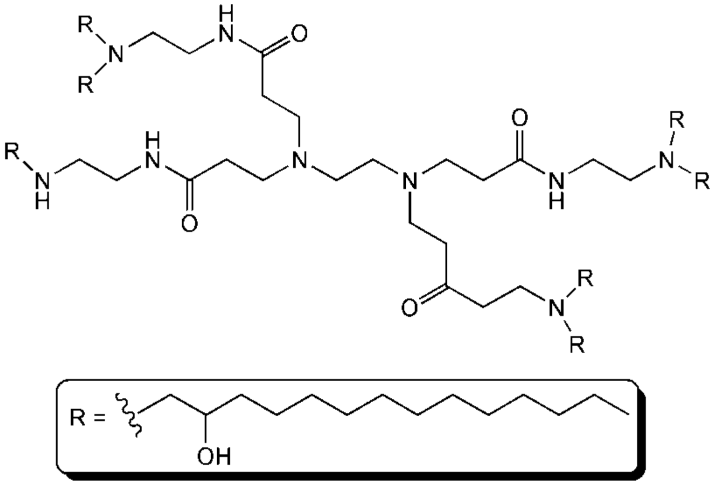

Figure S14. Chemical structure of amphiphilic cationic lipid-like compound G0-C14.

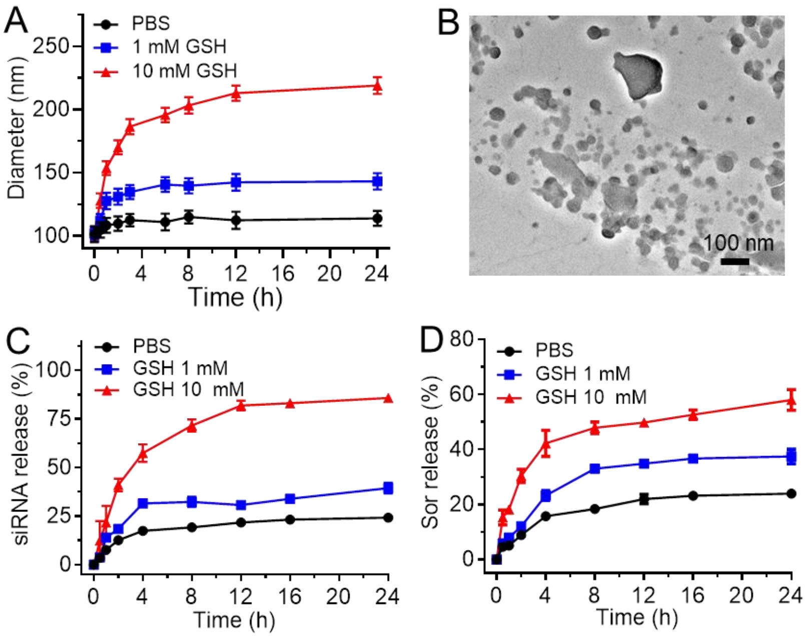

Figure S15. (A) Size change of NPs(siCFL1/Sor) incubated in GSH-containing PBS solution for different time. (B) Morphology of NPs(siCFL1/Sor) incubated in PBS solution containing 10 mM GSH for 24 h. (C, D) Cumulative siCFL1 (C) and sorafenib release (D) from the NPs(siCFL1/Sor) incubated in GSH-containing PBS solution for different time.

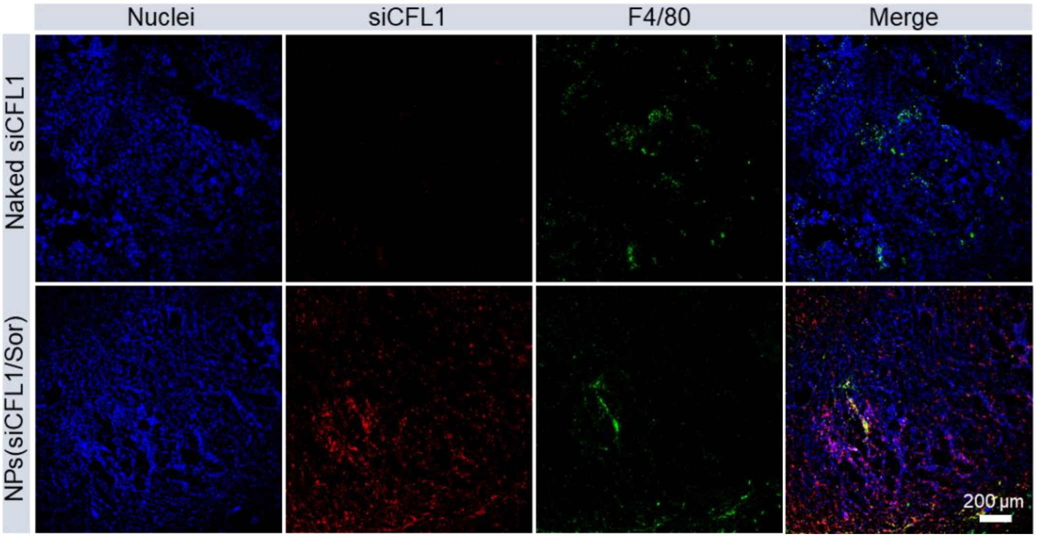

Figure S16. IF analysis of the tumor tissues of tumor-bearing mice sacrificed at 24 h post injection of naked siCFL1 and NPs(siCFL1/Sor).

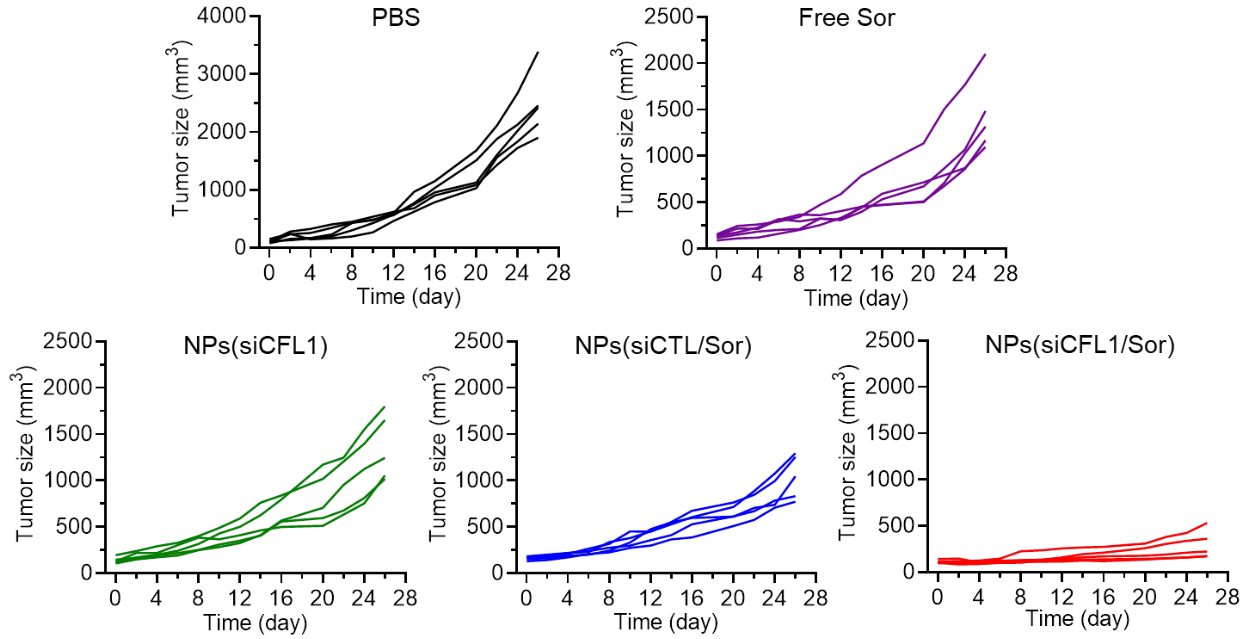

Figure S17. Tumor size of MHCC-97L xenograft tumor-bearing mice treated with PBS, free sorafenib (Free Sor), NPs(siCFL1), NPs(siCTL/Sor), and NPs(siCFL1/Sor).

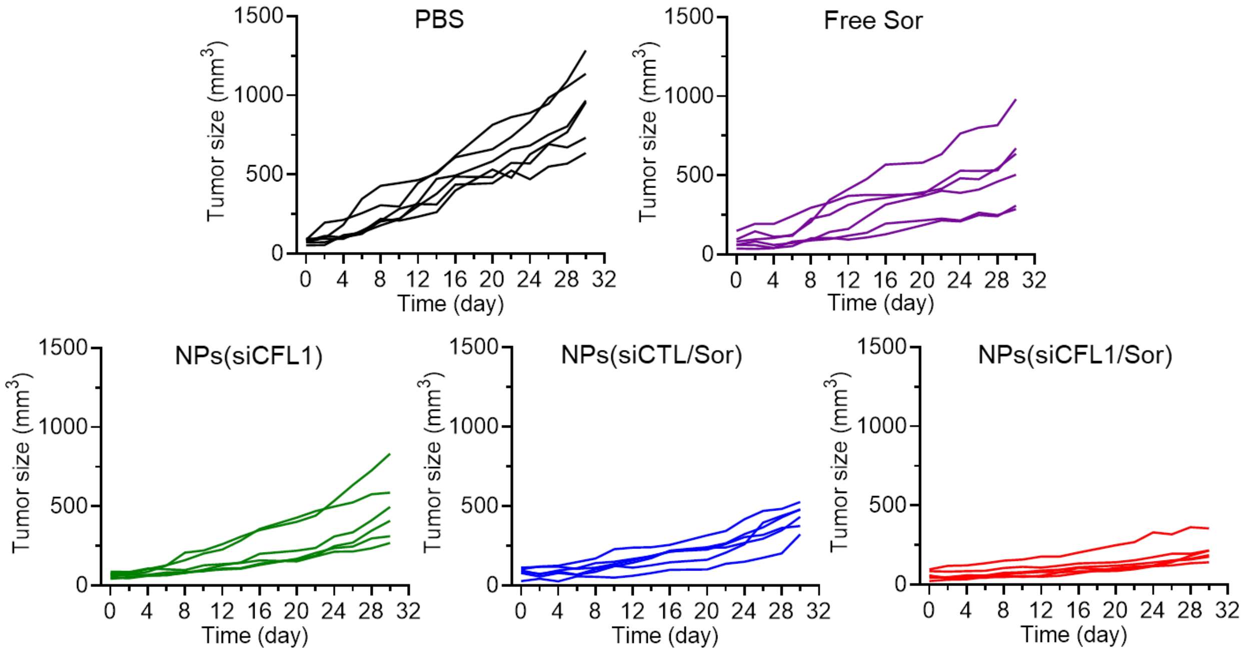

Figure S18. Tumor size of PDX tumor-bearing mice treated with PBS, free sorafenib (Free Sor), NPs(siCFL1), NPs(siCTL/Sor), and NPs(siCFL1/Sor).

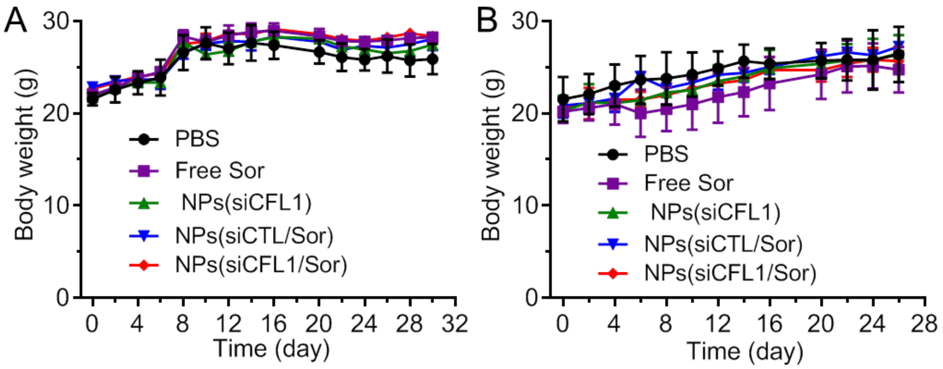

Figure S19. Body weight of MHCC-97L xenograft (A) and PDX (B) tumor-bearing mice treated with PBS, free sorafenib (Free Sor), NPs(siCFL1), NPs(siCTL/Sor), and NPs(siCFL1/Sor).

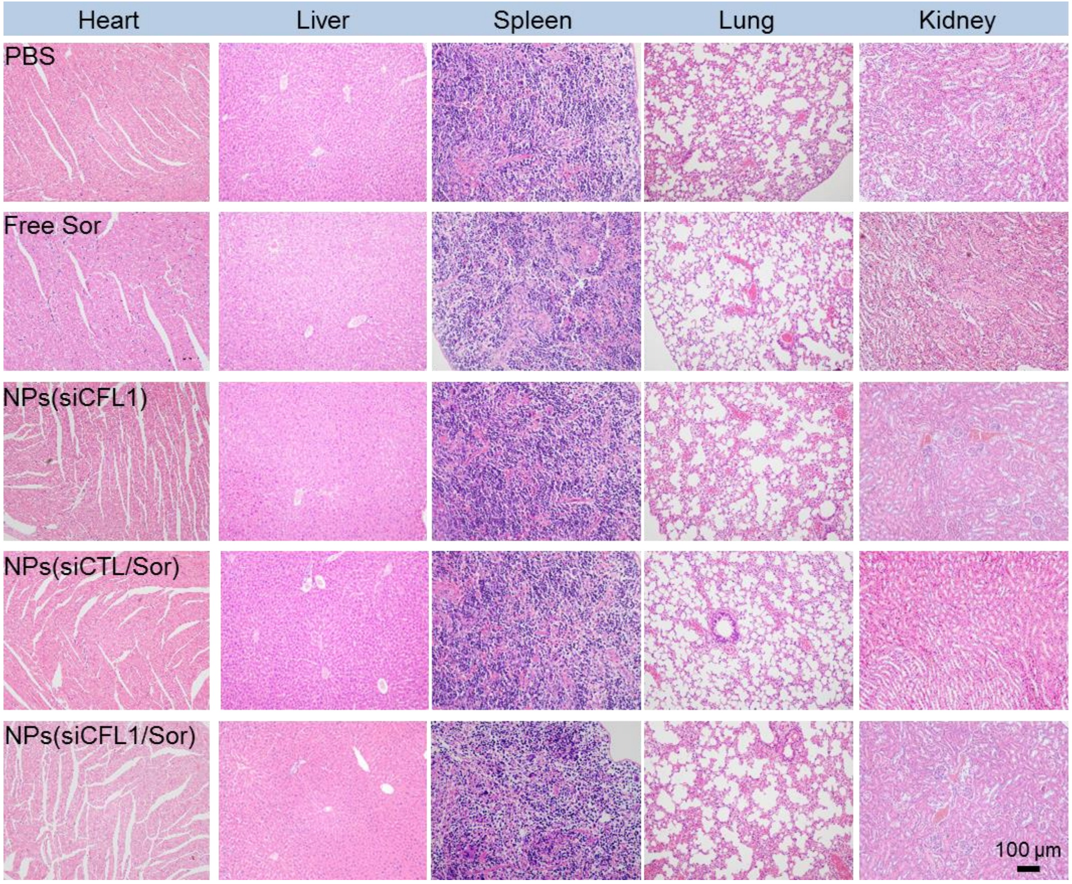

Figure S20. Histological analysis of the major organs of healthy mice received three consecutive intravenous injections of PBS, free sorafenib (Free Sor), NPs(siCFL1), NPs(siCTL/Sor), and NPs(siCFL1/Sor).

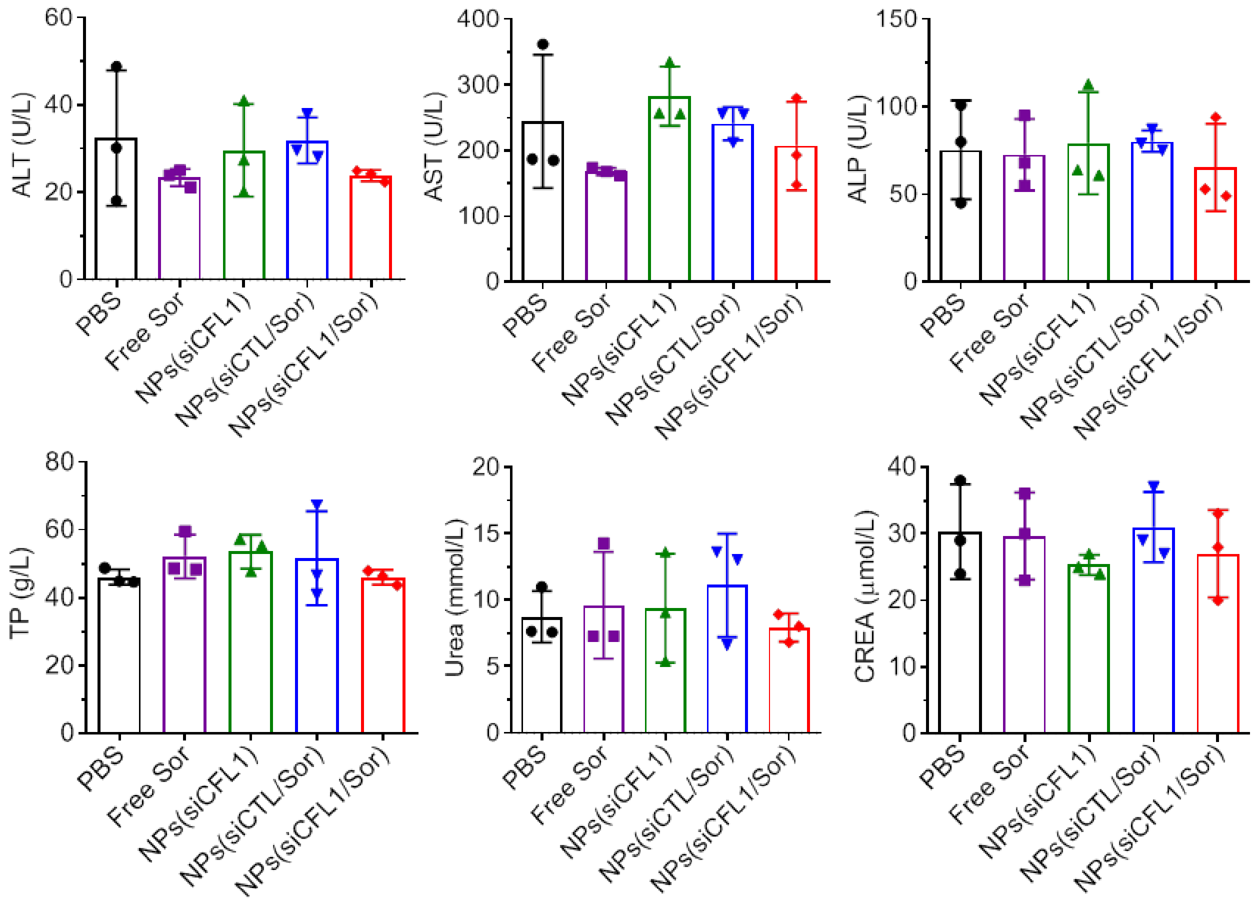

Figure S21. Serum levels of alanine aminotransferase (ALT), aspartate aminotransferase (AST), alkaline phosphatase (ALP), total protein (TP), Urea, and creatinine (CREA) of healthy mice received three consecutive intravenous injections of PBS, free sorafenib (Free Sor), NPs(siCFL1), NPs(siCTL/Sor), and NPs(siCFL1/Sor).
